# Randomized Controlled Trial of Zoledronic Acid plus Chemotherapy versus Chemotherapy Alone as Neoadjuvant Treatment of HER2-Negative Primary Breast Cancer (JONIE Study)

**DOI:** 10.1371/journal.pone.0143643

**Published:** 2015-12-03

**Authors:** Yoshie Hasegawa, Hirokazu Tanino, Jun Horiguchi, Daishu Miura, Takashi Ishikawa, Mitsuhiro Hayashi, Shintaro Takao, Seung Jin Kim, Kazuhiko Yamagami, Masaru Miyashita, Muneharu Konishi, Yasushi Shigeoka, Masato Suzuki, Tetsuya Taguchi, Tomoyuki Kubota, Kouhei Akazawa, Norio Kohno, The JONIE Study Group

**Affiliations:** 1 Department of Breast Surgery, Hirosaki Municipal Hospital, Aomori, Japan; 2 Department of Breast and Thyroid Surgery, Kitasato University Hospital, Kanagawa, Japan; 3 Department of Breast and Endocrine Surgery, Gunma University Hospital, Gunma, Japan; 4 Department of Breast and Endocrine Surgery, Toranomon Hospital, Tokyo, Japan; 5 Department of Breast and Thyroid Surgery, Yokohama City University Medical Center, Kanagawa, Japan; 6 Department of Breast Oncology, Tokyo Medical University Hachioji Medical Center, Tokyo, Japan; 7 Department of Breast Surgery, Hyogo Cancer Center, Hyogo, Japan; 8 Department of Breast and Endocrine Surgery, Graduate School of Medicine, Osaka University, Osaka, Japan; 9 Department of Breast Oncology, Shinko Hospital, Hyogo, Japan; 10 Department of Surgery, Konan Hospital, Hyogo, Japan; 11 Department of Surgery, Hyogo Prefectural Nishinomiya Hospital, Hyogo, Japan; 12 Department of Medical Oncology, Yodogawa Christian Hospital, Osaka, Japan; 13 Department of Surgery, Teikyo University Chiba Medical Center, Chiba, Japan; 14 Department of Endocrine & Breast Surgery, Kyoto Prefectural University of Medicine, Kyoto, Japan; 15 Department of Breast Surgery, Kamiiida Daiichi General Hospital, Nagoya, Japan; 16 Department of Medical Informatics, Niigata University Medical and Dental Hospital, Niigata; 17 Department of Breast Oncology, Kobe Kaisei Hospital, Hyogo, Japan; University Campus Bio-Medico, ITALY

## Abstract

**Purpose:**

Zoledronic acid (ZOL) is a nitrogen-containing bisphosphonate that induces osteoclast apoptosis and inhibits bone resorption by inhibiting the mevalonate pathway. Its benefit for the prevention of skeletal complications due to bone metastases has been established. However, the antitumor efficacy of ZOL, although suggested by multiple preclinical and clinical studies, has not yet been clinically proven. We performed the present randomized Phase 2 trial to investigate the antitumor effect of ZOL with chemotherapy (CT).

**Methods:**

Asian patients with HER2-negative invasive breast cancer were randomly assigned to either the CT or CT+ZOL (CTZ) group. One hundred and eighty-eight patients were randomized to either the CT group (*n* = 95) or the CTZ group (*n* = 93) from March 2010 to April 2012, and 180 patients were assessed. All patients received four cycles of FEC100 (fluorouracil 500 mg/m^2^, epirubicin 100 mg/m^2^, and cyclophosphamide 500 mg/m^2^), followed by 12 cycles of paclitaxel at 80 mg/m^2^ weekly. ZOL (4 mg) was administered three to four times weekly for 7 weeks to the patients in the CTZ group. The primary endpoint was the pathological complete response (pCR) rate, which was defined as no invasive cancer in the breast tissue specimen. Safety was assessed in all patients who received at least one dose of the study drug.

**Results:**

This randomized controlled trial indicated that the rates of pCR in CTZ group (14.8%) was doubled to CT group (7.7%), respectively (one-sided chi-square test, p = 0.068), though the additional efficacy of zoledronic acid was not demonstrated statistically. The pCR rate in postmenopausal patients was 18.4% and 5.1% in the CTZ and CT groups, respectively (one-sided Fisher’s exact test, p = 0.071), and that in patients with triple-negative breast cancer was 35.3% and 11.8% in the CTZ and CT groups, respectively (one-sided Fisher’s exact test, p = 0.112). Thus the addition of ZOL to neoadjuvant CT has potential anticancer benefits in postmenopausal patients and patients with triple-negative breast cancer. Further investigation is warranted.

**Trial Registration:**

University Hospital Medical Information Network. UMIN000003261.

## Introduction

Bisphosphonates have a high affinity for hydroxyapatite on the bone surface and inhibit osteoclastic bone resorption [1]. Zoledronic acid (ZOL) is a nitrogen-containing bisphosphonate that powerfully suppresses bone resorption. It has been clinically developed to prevent skeletal morbidity secondary to bone metastasis as well as to treat osteoporosis [2]. In 2005, Kohno *et al*. [3] performed a randomized placebo-controlled clinical trial of ZOL in patients with metastasis of breast cancer to bone and reported both an analgesic effect and significant reduction in the frequency of skeletal-related events. Furthermore, ZOL seems to have antitumor efficacy. In *in vitro* experimental systems, ZOL reportedly enhances apoptotic effects when used concomitantly with anticancer agents. Antitumor effects of ZOL in combination with antitumor agents *in vivo* have also been reported [4]. In one study, clinical recurrence of breast cancer was reportedly less frequent when ZOL was added to postoperative adjuvant endocrine therapy for premenopausal patients with breast cancer receiving ovarian suppression therapy [5]. In another study, although the efficacy of ZOL did not improve when used in combination with chemotherapy (CT), benefits were seen in the subset of women who were postmenopausal at the time of diagnosis [6]. Clinical trials have reported conflicting results on whether oral clodronic acid therapy improves survival in patients with breast cancer. One phase-III trial that enrolled 3323 women with stage I to III breast cancer did not show a significant reduction in recurrence [7,8]. Thus, the anticancer efficacy of bisphosphonates has not yet been clinically proven.

In the present study, we attempted to demonstrate the efficacy of concomitant ZOL and CT in patients with operable HER2-negative breast cancer who received neoadjuvant CT comprising FEC100 followed by weekly paclitaxel. The effect of additional ZOL was measured using the pathological complete response (pCR) rate as the primary endpoint. A relatively higher pCR rate, particularly visible in a few subgroups, was observed in the concomitant ZOL group.

## Methods

### Study design and patients

The patients in this study were recruited from 16 sites of the Japan Organization of Neoadjuvant Innovative Experts (JONIE) trial group in Japan. Eligible patients were those with histologically proven invasive breast cancer of clinical stage IIA to IIIB (T ≥ 3.0 cm and node-negative, or T ≥ 2.0 cm and cytologically or pathologically defined node-positive) and HER2-negative (HER2 0 or 1+ on immunohistochemistry, or 2+ on immunohistochemistry and negative on fluorescence *in situ* hybridization). All patients were newly diagnosed by core needle biopsy from 20 to 70 years of age; all had an Eastern Cooperative Oncology Group performance status of 0–1 and normal cardiac, renal (creatinine clearance rate of ≥60 mL/min), and liver functions. The exclusion criteria were bilateral breast cancer or inflammatory breast cancer; distant metastasis; a history of chemotherapy, endocrine therapy, or radiotherapy for breast cancer; serious comorbidities such as heart failure, cardiac infarction, or serious disorders of infection; a complicating dental or jaw infection or traumatic condition of the teeth; and a history of treatment with a bisphosphonate within the previous 12 months.

The protocol, including the documentation of informed consent and patient information, was approved by the independent ethics committee at each participating site (See S1 Protocol). The study was performed in accordance with the International Conference on Harmonisation guidelines concerning Good Clinical Practice and the Declaration of Helsinki. The study investigators provided an information form approved by each institutional review board to all patients before enrollment to explain the following, and obtain voluntary written informed consent to participate in the study from the patient.

### Randomization and masking

This was a multicenter, open-label, randomized phase-II trial. All eligible patients were randomly assigned, in a 1:1 ratio, to either the experimental CT + ZOL group (CTZ group) or the standard CT-alone group (CT group). Randomization was centralized at the data center of JONIE trial group (Niigata University, Japan) and performed using the minimization method. Patients were stratified according to institution, lymph node metastasis (presence versus absence), estrogen receptor status (positive versus negative), and menopausal status (premenopausal versus postmenopausal). Patients aged >55 years who had been amenorrheic for 1 year were determined to be postmenopausal. The status of younger amenorrheic patients was determined by measurement of follicle-stimulating hormone, luteinizing hormone, and estradiol levels.

Masking of the patients, investigators, and data analysts was not feasible because of the nature of the two different dosing regimens.

### Treatment protocol

Four cycles of FEC100 (fluorouracil 500 mg/m^2^, epirubicin 100 mg/m^2^, and cyclophosphamide 500 mg/m^2^) was administered by intravenous (iv) infusion every 3 weeks followed by 12 cycles of paclitaxel at 80 mg/m^2^ by iv infusion once weekly. ZOL (4 mg) was administered by iv infusion four times every 3 weeks with every administration of FEC100 and three times every 4 weeks during every administration of paclitaxel in the CTZ group. Dose reductions of epirubicin from 100 to 75 mg/m^2^ and of paclitaxel from 80 to 60 mg/m^2^ were permitted in patients with febrile neutropenia and grade 3 or 4 nonhematological toxicities. Adverse events were graded using the National Cancer Institute Common Terminology Criteria for Adverse Events (CTCAE) version 3.0.

All patients underwent a complete blood count with differential and serum chemistry analysis before every CT cycle, and breast ultrasound or caliper assessment was performed at the planned times at weeks 6, 12, 16, 20, and 24 of CT. Magnetic resonance imaging or computed tomography was performed within 28 days before enrollment and at weeks 12 and 24, between the two regimens and at the end of CT. After surgery, the patients visited the hospital for follow-up every 3 months.

After CT, following the above-described protocol, the following data were recorded: surgical procedures, pathological diagnosis, and clinical response based on ultrasonography, magnetic resonance imaging, or computed tomography using the RECIST criteria. The central review of the surgical specimens was performed in the Department of Pathology of Yokohama City University, Yokohama, Japan and included measurement of estrogen, progesterone, and HER2 receptor expression as well as pathological response to the CT with or without ZOL. All pathologists were blinded to the treatment regimen.

The primary endpoint was the pCR rate, which was defined according to the NSABP guidelines as no invasive cancer in the surgical breast tissue specimens. The primary endpoint(pathological complete response) was centrally reviewed.

The secondary endpoints were the clinical response rates (complete response [CR] and partial response [PR] rates) based on RECIST ver. 1.0, the rate of breast-conserving surgery, disease-free survival (DFS) (defined as the time from randomization to disease occurrence or death), and safety.

### Statistical analysis

The endpoints were analyzed following an intention-to-treat principle. Exploratory subset analyses were subsequently performed using the patients in some strata. Based on previous studies [6,8], we assumed that the pCR rate would be 25%. We thus estimated that 80 evaluable patients should be enrolled in each group to achieve a 20% increase in the pCR rate in the CTZ group with a power of 80% at a significance level of 0.05 in a one-sided chi-squared test.

Descriptive analyses were used to summarize the data. Median and [minimum, maximum] were used for continuous variables, and frequency counts and percentages were used for categorical variables.

The chi-squared and Fisher’s exact tests were used to test the null hypothesis that the proportions of categories are equal between the CT and CTZ groups. The Mann–Whitney test was used to compare the distributions of continuous or ordered variables between the two groups. The level of statistical significance was set at 0.05 (one-sided). All analyses were performed using the statistical software SPSS, version 17.0 (SPSS, Inc., Chicago, IL, USA).

This study was registered at the University Hospital Medical Information Network as UMIN000003261 (www.umin.ac.jp/english/).

## Results

### Patient characteristics

**Fig 1:**
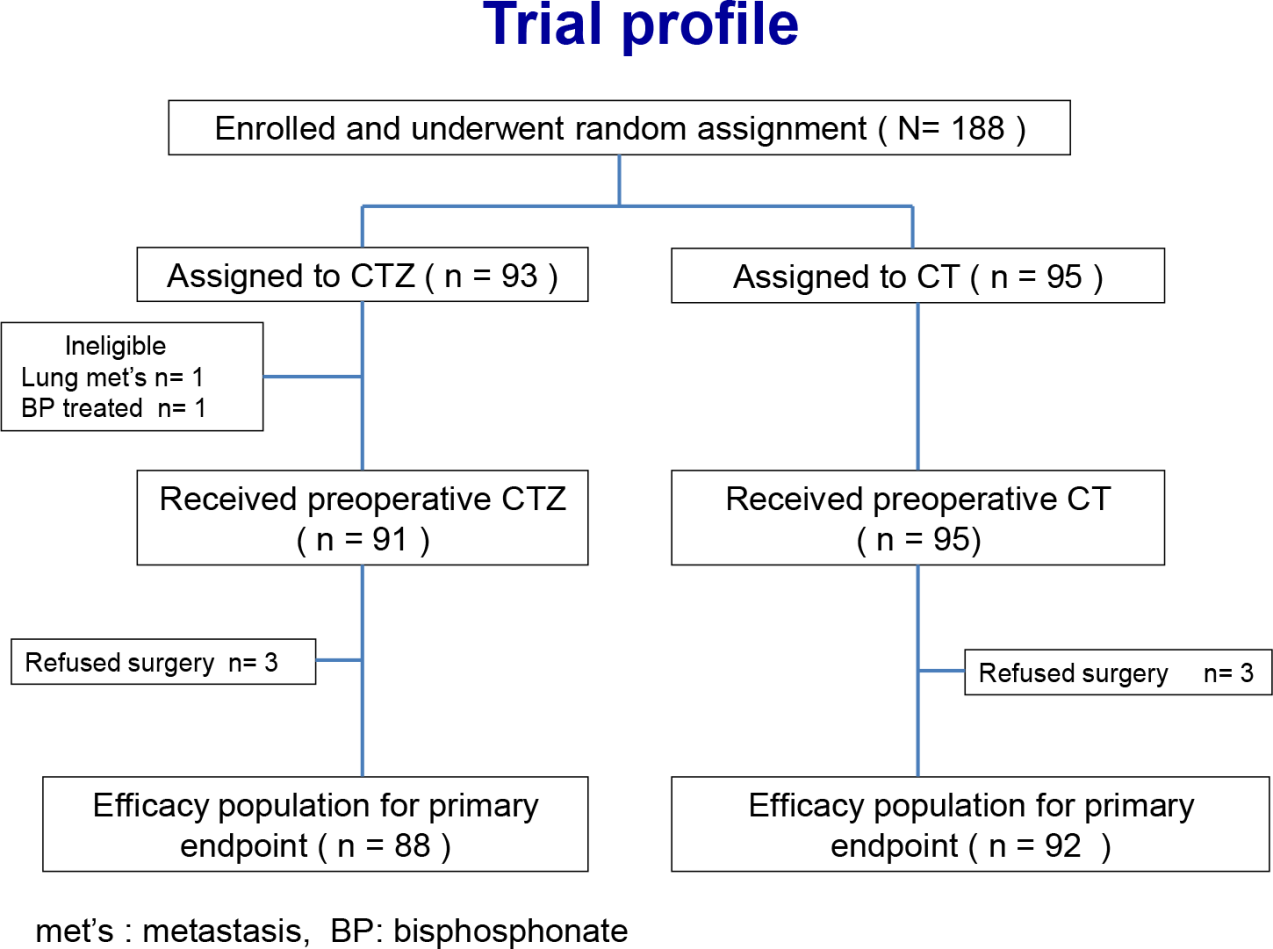
CONSORT diagram for JONIE Study.

From March 2010 to June 2012, 188 patients were randomly assigned to either the CTZ group (*n* = 93) or the CT group (*n* = 95). Two patients in the CTZ group were ineligible because one was diagnosed with lung metastasis and the other had a history of bisphosphonate treatment. Among the 186 eligible patients, three in the CTZ group and three in the CT group refused surgery and were thus excluded from the intention-to-treat analyses (Fig 1). The patient characteristics are shown in Table 1. The median age was 49.5 years (range, 34–71 years) in the CTZ group and 49.0 years (range, 28–70) in the CT group. The proportion of postmenopausal patients in the CTZ and CT group was 43.2% and 42.3%, respectively. The most frequent tumor size category was clinical T2. In total, 34.1% of patients in the CTZ group and 37.0% in the CT group were pathologically node-positive. The frequency of triple-negative intrinsic subtypes in the CTZ and CT group was 19.3% and 18.5%, respectively.

**Table 1:** Patients’ characteristics.

	CTZ Group n = 88(%)	CT Group n = 92(%)	p-value*
**Age at randomization**			
mean	49.5	49	0.912
range	34–71	28–70	
**Menopausal status**			
Premenopausal	50(56.8)	53(57.6)	1.000
Postmenopausal	38(43.2)	39(42.4)	
**T-stage**			
cT1	2(2.3)	1(1.1)	0.784
cT2	66(75.0)	73(79.3)	
cT3	13(14.8)	13(14.1)	
cT4	7(8.0)	5(5.4)	
**N-stage**			
pN+	30(34.1)	34(37.0)	0.756
pN-	58(65.9)	58(63.0)	
**ER status**			
ER+	71(80.7)	75(81.5)	1.000
ER-	17(19.3)	17(18.5)	
**PgR status**			
PgR+	66(75.0)	69(75.0)	1.000
PgR-	22(25.0)	23(25.0)	
**Histology**			
Invasive ductal carcinoma	85(96.6)	88(95.7)	1.000
Invasive lobular carcinoma	1(1.1)	2(2.2)	
Others	2(2.3)	2(2.2)	
**Planned surgery**			
Lumpectomy	38(43.2)	41(44.6)	0.881
Mastectomy	50(56.8)	51(55.4)	

*Mann–Whitney U test and Fisher’s exact test (two-sided).

CTZ = chemotherapy + zoledronic acid; CT = chemotherapy alone; pN = pathological node-positive or -negative; ER = estrogen receptor; PgR = progesterone receptor.

Fourteen (15.3%) of the patients in the CTZ group failed to complete the trial (adverse events, *n* = 7; opted out of the trial, *n* = 3; disease progression, *n* = 1; requirement for dental surgery, *n* = 1; and refused surgery, *n* = 2). Eleven (11.6%) of the patients in the CT group were excluded (adverse events, *n* = 6; preferred treatment without paclitaxel, *n* = 2; clinical disease progression, *n* = 1; and refused surgery, *n* = 2). One patient who inadvertently received triweekly docetaxel rather than weekly paclitaxel was included in the response rate and safety analyses upon termination of FEC. The pCR rate among all patients was 11.2%.

### pCR rate

The pCR rate (Table 2) was 14.8% (13/88 patients) and 7.7% (7/91 patients) in the CTZ and CT groups, respectively (p = 0.068; one-sided chi-squared test). The pCR rates in the postmenopausal patients were 18.4% and 5.1% in the CTZ group (*n* = 38) and CT group (*n* = 39), respectively (p = 0.071; Fisher’s exact test). The pCR rates in patients with triple-negative breast cancer were 35.3% and 11.8% in the CTZ group (*n* = 17) and CT group (*n* = 17), respectively (p = 0.112; Fisher’s exact test). Finally, the pCR rates in both the postmenopausal patients and patients with triple-negative breast cancer were 50.0% and 0.0% in the CTZ group (*n* = 8) and CT group (*n* = 9), respectively (p = 0.029; Fisher’s exact test).

**Table 2:** Pathological complete response.

	TOTAL	CTZ group(%)95%CI^†^		CT group(%) 95%CI^†^		Pvalue*
All patients	179	13/88(14.8)	7.4–22.2	7/91(7.7)	2.2–13.2	0.066
Pre menopausal	102	6/50(12.0)	3.0–21.0	5/52(9.6)	1.6–17.6	0.349
Post menopausal	77	7/38(18.4)	6.1–30.7	2/39(5.1)	0.0–12.0	0.071**
Luminal	145	7/71(9.9)	3.0–16.8	5/74(6.8)	1.1–12.5	0.249
Triple negative (TN)	34	6/17(35.3)	12.6–58.0	2/17(11.8)	0.0–27.1	0.112**
Pre menopausal & TN	17	2/9 (22.2)	0.0–49.1	2/8(25.0)	0.0–55.0	0.665**
Post menopausal & TN	17	4/8(50.0)	15.4–84.6	0/9 (0.0)		0.029**

† 95 percent confidence interval.

*P value based on one-sided chi-squared test.

**P value based on Fisher’s exact test.

CTZ = chemotherapy + zoledronic acid; CT = chemotherapy alone.

### Clinical response and breast-conserving surgery

The clinical response rates were 83.1% (19.8% CR and 63.9% PR) and 82.6% (17.4% CR and 65.1% PR) in the CTZ and CT groups, respectively (p = 0.407; Mann–Whitney U test) (Table 3). However, five patients in the CTZ group and six patients in the CT group were excluded from this analysis because of insufficient information on physical examination after CT.

**Table 3:** Adverse events (grade ≥ 3).

Adverse event	CTZ Group (n = 91)	CT Group (n = 95)	p-value*
WBC decreased	38 (41.8)	30 (31.6)	0.075
Neutrophil count decreased	39 (42.9)	34 (35.8)	0.162
Febrile neutropenia	13 (14.3)	14 (14.7)	0.465
Sensory neuropathy	8 (8.8)	5 (5.3)	0.173

*P value based on one-sided chi-squared test.

CTZ = chemotherapy + zoledronic acid; CT = chemotherapy alone.

After the systemic treatment, 180 (96.8%) patients underwent surgery (Table 1). Breast-conserving surgery was possible in 45 (51.1%) of the 88 patients treated with ZOL and in 40 (43.5%) of the 92 patients treated with CT (p = 0.152; one-sided chi-squared test).

### Safety

The proportions of grade ≥3 adverse events shown in Table 3. The white blood cell counts in the CTZ and CT groups were low at 43.2% and 32.6%, respectively, and the neutrophil counts were low at 44.3% and 37.0%, respectively. There were no significant differences in the proportions of severe toxicities (grade 3–4) between the two groups. In total, 88 patients in the CTZ group and 92 in the CT group completed the CT (data not shown).

## Discussion and Conclusions

While the bone-modifying mechanism of ZOL has been explained, the mechanism of its antitumor efficacy has not been fully elucidated. Previous studies have suggested a direct antitumor effect of ZOL and synergistic effect in combination with anticancer agents [9]. However, there was not predetermined chemotherapy regimen, and it contained HER2 positive patients. Therefore, we planned a new trial to define additional efficacy of zoledronic acid onto standard neo-adjuvant chemotherapy using FEC100 followed by weekly paclitaxel for HER2 negative breast cancer. This randomized controlled trial suggested the negative result regarding the additional efficacy of zoledronic acid, though the rates of pCR in CTZ (14.8%) group was doubled to CT group (7.7%). It was due to over-optimistic assumptions of the power analysis. Improved efficacy has been achieved *in vitro* by adding ZOL to standard adjuvant chemotherapy agents, FEC or TAC, for the treatment of breast cancer [10]. These results led to the present JONIE trial, which attempted to elucidate the clinically relevant antitumor effects of ZOL. Although not quite significant at the 0.05 level, with the relatively higher pCR rate, concomitant ZOL seems to show clinically beneficial antitumor effect on at least some of the breast cancer patients who receive neoadjuvant CT. The exploratory subset analyses of the JONIE Study suggest that postmenopausal and/or triple-negative breast cancer patients could be the most promising group to be effectively treated with additional ZOL.

Several trials of the neoadjuvant use of ZOL have been performed. As for postmenopausal breast cancer, the JONIE trial results is consistent with those of the NEOZOTAC, which showed a small benefit of ZOL added to CT against postmenopausal breast cancer [11]. On the other hand, the results for premenopausal patients turned out to be mixed; unlike the NEOZOTAC trial, in which the pCR rate was lower in the concomitant ZOL group among the premenopausal patients with breast cancer, the pCR rate was improved, albeit slightly, among the premenopausal patients with breast cancer treated with ZOL in the JONIE trial.

The JONIE results also support a previous investigation of the antitumor effects of ZOL according to cancer subtypes. Aft et al. [12] evaluated the antitumor efficacy of ZOL by detecting disseminated tumor cells in bone marrow samples, and reported a significant benefit of adding ZOL to the treatment of triple-negative breast cancer [12]. Similarly, in the analysis of the HR–/ HER2– subtype group, the JONIE trial revealed a higher pCR rate in the patients treated with ZOL (35.3% in the CTZ group and 11.8% in the CT group; p = 0.112). Among the patients with HR+ and HER2– breast cancer, the pCR rate was slightly higher in the CTZ group (9.9%) than in the CT group (6.8%; p = 0.249). CT with ZOL could be a promising option for the treatment of triple-negative breast cancer.

Although the scope of the present study is limited to CT, studies suggest ZOL can also be effective when added to endocrine therapy. In the ABCSG12 trial, concomitant ZOL was associated with significantly better disease-free and overall survival than was adjuvant endocrine therapy comprising luteinizing hormone-releasing hormone agonists plus tamoxifen or anastrozole in 1803 premenopausal patients with breast cancer [5, 13]. The researchers of AZURE trial, a two-group study that evaluated the effects of ZOL added to adjuvant endocrine therapy or CT, reported that more frequent metastases to organs other than the bones were found in the premenopausal, perimenopausal, and unknown menopausal patients in the ZOL combination group [14]. A subset of postmenopausal patients in the ZOL group showed a better DFS rate [15]. The ZO-FAST trial, which involved 1065 postmenopausal patients with breast cancer, demonstrated better DFS with the addition of ZOL to adjuvant endocrine therapy with letrozole [16]. Thus, if the estrogen level is low, ZOL in combination with either endocrine therapy or CT may be effective.

ZOL is a safe agent when administered intravenously, with no serious side effects other than occasional osteonecrosis of the jaw. It is approved for the treatment of osteoporosis in postmenopausal women and can effectively suppress breast cancer recurrence and metastasis. ZOL, however, has not been approved in any countries as adjuvant therapy of breast cancer, partly because in spite of abundant positive data, including the results of a meta-analysis reported in 2013 [17], its mechanism of action is unknown. The present study warrants that the efficacy of ZOL should be verified in further clinical trials with the goal of gaining approval by regulatory and health insurance agencies. Additionally, the development of biomarkers is indispensable for the identification of effective targets. A robustly designed clinical trial of ZOL in combination with neoadjuvant CT for the treatment of postmenopausal triple-negative breast cancer would enable our preliminary observations to be formally tested.

## Supporting Information

S1 CONSORT Checklist CONSORT checklist for JONIE Study. (DOC)

S1 Protocol Original protocol. (DOC)
