# Supplementary material for: Randomized Controlled Trial of Zoledronic Acid plus Chemotherapy versus Chemotherapy Alone as Neoadjuvant Treatment of HER2-Negative Primary Breast Cancer (JONIE Study)
Source: PLoS One. 2015 Dec 3;10(12):e0143643. doi: 10.1371/journal.pone.0143643 (PMC4669153; doi:10.1371/journal.pone.0143643)
Supplement: S1 Protocol — (DOC) [file pone.0143643.s002.doc]

***JONIE***

Japan Organization of Neoadjuvant Innovative Expert

Randomized controlled trial comparing zoledronic acid plus chemotherapy with chemotherapy alone as neoadjuvant therapy in patients with primary breast cancer

Protocol

Principal investigator: Norio Kohno

Department of Breast Oncology, Tokyo Medical University Hospital

Nishisinjuku 6-7-1, Shinjuku-ku,Tokyo, 160-0023

TEL: 03-3342-6111

E-mail: [nkohno@tokyo-med.ac.jp](mailto:nkohno@tokyo-med.ac.jp)

Study administrative office: Hiroshi Kaise

Department of Breast Oncology, Tokyo Medical University Hospital

Nishisinjuku 6-7-1, Shinjuku-ku, Tokyo, 160-0023

TEL: 03-3342-6111

E-mail: kaise3632@yahoo.co.jp

Protocol ver. 1.0: 19 November 2009

Statistical analysis plan revised on: 23 February 2010

**0. Protocol Synopsis**

0.1. Schema

Patients with preveiously untreated stage IIA to IIIB primary breast cancer

Tumor diameter of 3 cm or more and axillary lymph node metastasis +

HER 2 (−), ECOG PS: 0-1

age >20to ≤ 70 years

Obtain informed consent and enroll the patient

Randomization

(1) Presence or absence of lymph node metastasis

(2) ER expression (3) Menopausal status

FEC100 every 3 weeks for 4 cycles

→Weekly PTX 60 mg/m^2^ for 12 cycles^*1^

(Chemotherapy alone group)

FEC100 every 3 weeks for 4 cycles

→Weekly PTX 80 mg/m^2^ for 12 cycles^*1^

+

ZOL (7 times at an interval of 3 to 4 weeks)

*1: Weekly PTX 80 mg/m^2^ for 12 cycles → FEC100 every 3 weeks for 4 cycles is also allowed.

0. 2. Objective

To evaluate the efficacy and safety of neoadjuvant chemotherapy in combination with zoledronic acid in patients with stage IIA to IIB HER2-negative primary breast cancer

0. 2-1. Primary endpoint

Pathological complete response rate: pCR*

* According to the definition of NSABP B-18 study, pathological complete response means “the absence of invasive disease in the breast, irrespective of the presence of any residual disease in the axillary nodes.”

0. 2-2. Secondary endpoints

1. Tumor response rate, which is defined as the percentage of patients who have achieved complete response (CR) and partial response (PR)

2. Proportion of breast-conserving surgery

3. Disease free survival rate

4. Adverse events

0. 3. Study population

0. 3.1. Inclusion criteria

1. Women who have histologically proven invasive breast cancer

2. Patients with clinical stage 2A to 3B and if any of the following criteria applies:

1) Tumor measuring 3 cm or more in diameter

2) Tumor measuring 2 cm or more in diameter and axillary lymph node metastasis (N1) is confirmed by biopsy or cytology.

3. Immunohistochemistry (IHC) score of 0 or 1+ by Hercep Test. If the score is 2+, the patients need to be proven HER2-negative by fluorescence *in situ* hybridization.

4. Patients who are ≥ 20 to ≤ 70 years of age at the time of enrollment

5. Patients with ECOG performance status of 0 or 1

6. Patients who have normal organ function as described below, in principle, within 14 days before enrollment

1) WBC ≥ 3,000 /mm^3^ and Neutrophils ≥ 1,500 /mm^3^

2) Hb ≥ 9.0 g/dL

3) Platelets ≥ 100,000 /mm^3^

4) AST or ALT ≤ 2.5 x upper limits of normal (ULN) defined by each study site

5) Serum Creatinine ≤ 1.5 mg/dl

6) Normal electrocardiogram

7) Echocardiography: left ventricular ejection fraction (EF) ≥ 50%

7. Patients who have provided written informed consent

0. 3.2. Exclusion criteria

1. Patients with non-invasive breast cancer or invasive micropapillary carcinoma

2. Patients with inflammatory breast cancer

3. Patients with bilateral breast cancer including metachronous contralateral breast cancer

4. Patients having previously received treatment of breast cancer such as chemotherapy, hormone therapy or radiation therapy

5. Patients with active double cancer

6. Patients who have a history or comorbidity of cardiac conditions described below:

(1) a history of myocardial infarction or congestive heart failure

(2) a comorbidity of ischemic cardiac disease, arrhythmias, myocardial damage or valvular disorder

7. Patients with serious complications including infectious diseases, pulmonary fibrosis, interstitial pneumonia, peripheral neuropathy, uncontrolled diabetes, and bleeding tendency

8. Patients who are pregnant or breastfeeding, or of childbearing potential

9. Patients who have dental problems including infection of the teeth or jawbone and dental trauma, or those who are scheduled for dental or jaw surgery within the next 6 weeks (e.g., extraction, implants)

10. Patients who have viral hepatitis, and those who are HBs antigen-positive or HCV antibody-positive

11. Patients who have received bisphosphonate in the past 12 months

12. Patients who are considered unsuitable by the investigators

0. 4. Treatment

0.4.1. Neoadjuvant therapy

5-FU 500 mg/m^2^, epirubicin 100 mg/m^2^ and CPA 500 mg/m^2^ (called FEC 100 treatment) will be administered every 3 weeks for 4 cycles, and followed by weekly paclitaxel 80 mg/m^2^ for 12 cycles.

Paclitaxel 80 mg/m2 may be first administered weekly for 12 cycles, and followed by FEC 100 every 3 weeks for 4 cycles. Neoadjuvant therapy regimen should be identical in each study site.

0.4.2. Zoledronic acid

Zoledronic acid 4 mg will be diluted in saline or 100 ml of 5% glucose and will be administered intravenously over 15 minutes.

Zoledronic acid will be administered 7 times at intervals of 3 to 4 weeks.

0.4.3. Assignment of Treatment Groups

At the time of enrollment, patients will be randomized to either treatment group A (CTZ group: chemotherapy + zoledronic acid) or B (CT group: chemotherapy alone) by the data center.

At the randomization, (1) the presence or absence of lymph node metastasis*, (2) ER expression (ER-positive cells ≥ 10%, or < 10%) and (3) menopausal status (premenopausal or postmenopausal) will be used as adjustment factors to minimize a bias between the treatment groups in a minimization method.

* Lymph node metastasis will be confirmed with imaging.

0.4.4. Surgery

After the completion of neoadjuvant chemotherapy, tumor response will be assessed by diagnostic imaging, and then an appropriate surgical procedure, such as breast-conserving surgery and mastectomy, will be performed based on the size and extent of the tumor.

Postoperative radiation therapy and hormone therapy in hormone-sensitive patients will be at the discretion of the investigator.

0.5. Planned sample size and study period

0.5.1. Planned sample size: 180 patients (90 patients in each group)

[Rationale for sample size calculation]

The pathological complete response (pCR) rate was 25% in a study of neoadjuvant chemotherapy with FEC followed by docetaxel in patients with primary breast cancer conducted by Toi et al.1)

The pCR rate of treatment with FEC100 followed by weekly paclitaxel in this study is estimated to be almost equal to that in the study by Toi et al.

While the pCR rate of neoadjuvant chemotherapy in AZURE study was 5.8% in the chemotherapy alone group, it was 10.9%, approximately 1.9-fold, in the zoledronic acid combination group. Similarly, if the pCR rate of FEC100 followed by weekly paclitaxel in combination with zoledronic acid is 1.9-fold of that of chemotherapy alone in this study, it will be 47.5%.

Therefore, assuming that the pCR rate is 25% in the chemotherapy alone (CT) group and 45% in the zoledronic acid combination (CTZ) group, a sample size of 80 patients in one group will be necessary to achieve the power of 80% with a one-sided significance level of 5%.

A total of 180 patients will be enrolled in both groups, including potential dropouts and withdrawals.

0.5.2. Study Period

Enrollment period: from March 2010 through June 2012

Study period: from January 2010 through December 2017, for 5 years after enrollment

Revision of the protocol is not required for an extension of the enrollment period of less than 6 months.

0.6. Contact Information

Central office:

Department of Breast Oncology, Tokyo Medical University Hospital

Hiroshi Kaise

**[Contents]**

[**0. Protocol Synopsis 1**](#__RefHeading___Toc423700531)

[**1. Objective 10**](#__RefHeading___Toc423700532)

[**1-1. Primary endpoint 10**](#__RefHeading___Toc423700533)

[**1-2. Secondary endpoints 10**](#__RefHeading___Toc423700534)

[**2. Background and Rationale for the Study 11**](#__RefHeading___Toc423700535)

[**2.1.　 Treatment of Breast Cancer and Preoperative (Neoadjuvant) Chemotherapy 11**](#__RefHeading___Toc423700536)

[**2.2.　 Clinical Study Results of Neoadjuvant Chemotherapy 11**](#__RefHeading___Toc423700537)

[**2.3.　Zoledronic Acid 12**](#__RefHeading___Toc423700538)

[**2.4.　 Treatment with Zoledronic Acid in Patients with Breast Cancer 13**](#__RefHeading___Toc423700539)

[**2.5.　 Zoledronic Acid as a Neoadjuvant Chemotherapy Drug in Patients with Breast Cancer 14**](#__RefHeading___Toc423700540)

[**2.6.　 Mechanism of Anti-tumor Activity of Bisphosphonates 14**](#__RefHeading___Toc423700541)

[**2.7.　 Rationale for the Study 14**](#__RefHeading___Toc423700542)

[**3. Criteria and Definitions in the Study 15**](#__RefHeading___Toc423700543)

[**3.1. Clinical Stage Classification 15**](#__RefHeading___Toc423700544)

[**3.2. Histological Classification 18**](#__RefHeading___Toc423700545)

[**3.3. Assessment of Change in Tumor Burden (Criteria for Clinical Response Evaluation) 19**](#__RefHeading___Toc423700546)

[**3.4. Histological Response Evaluation Criteria 20**](#__RefHeading___Toc423700547)

[**3.5. Performance Status (PS) 21**](#__RefHeading___Toc423700548)

[**3.6. Definition of Postmenopausal Patients 22**](#__RefHeading___Toc423700549)

[**3.7. Assessment of Recurrence 23**](#__RefHeading___Toc423700550)

[**4. Patient inclusion criteria 26**](#__RefHeading___Toc423700551)

[**4.1. Inclusion criteria 26**](#__RefHeading___Toc423700552)

[**5. Enrollment and Allocation 28**](#__RefHeading___Toc423700553)

[**5.1. Enrollment Procedure 28**](#__RefHeading___Toc423700554)

[**5.2. Considerations at Enrollment 28**](#__RefHeading___Toc423700555)

[**5.3. Randomization and Allocation Adjustment Factors 29**](#__RefHeading___Toc423700556)

[**6.1. Study Treatment 29**](#__RefHeading___Toc423700557)

[**6.2. Criteria for Study Treatment Discontinuation/Completion 30**](#__RefHeading___Toc423700558)

[**6.3. Criteria for Modification of Study Treatment 31**](#__RefHeading___Toc423700559)

[**6.4. Concomitant Therapy, Supportive Therapy 32**](#__RefHeading___Toc423700560)

[**6.5. Surgery 33**](#__RefHeading___Toc423700561)

[**6.6. Postoperative Therapy 34**](#__RefHeading___Toc423700562)

[**6.7. Follow-up after Surgery 34**](#__RefHeading___Toc423700563)

[**7.　 Potential Adverse Reactions 35**](#__RefHeading___Toc423700564)

[**7.1.　 Potential Adverse Reactions for Each Drug 35**](#__RefHeading___Toc423700565)

[**7.2. Assessment of Adverse Events/Adverse Reactions 35**](#__RefHeading___Toc423700566)

[**8. Endpoints, Laboratory Examinations, Evaluation Schedule 35**](#__RefHeading___Toc423700567)

[**8.1. Screening Tests Prior To Enrollment 35**](#__RefHeading___Toc423700568)

[**8.2. Tests and Evaluation During Study Treatment Period 36**](#__RefHeading___Toc423700569)

[**8.3. Tests and Evaluation After Completion of Study Treatment 37**](#__RefHeading___Toc423700570)

[**8.4.　 Study Schedule 39**](#__RefHeading___Toc423700571)

[**9. Data Collection 40**](#__RefHeading___Toc423700572)

[**9.1.　Case Report Form (CRF) 40**](#__RefHeading___Toc423700573)

[**10. Reporting of Adverse Events 40**](#__RefHeading___Toc423700574)

[**10.1. Adverse Events Requiring Reporting 40**](#__RefHeading___Toc423700575)

[**10.2. Reporting Requirements and Procedures for Site Principal Investigator 41**](#__RefHeading___Toc423700576)

[**11. Response Evaluation and Definition of Endpoints 43**](#__RefHeading___Toc423700577)

[**11.1. Pathological complete response rate (pCR)^＊^ 43**](#__RefHeading___Toc423700578)

[**11.2. Evaluation of Tumor Response 43**](#__RefHeading___Toc423700579)

[**11.3. Proportion of Breast-conserving Surgery 43**](#__RefHeading___Toc423700580)

[**11.5. Evaluation of Adverse Events 43**](#__RefHeading___Toc423700581)

[**12. Statistical Considerations 44**](#__RefHeading___Toc423700582)

[**12.1. Analysis Set 44**](#__RefHeading___Toc423700583)

[**12.2. Statistical Analysis Plan 44**](#__RefHeading___Toc423700584)

[**12.3. Planned Sample Size, Enrollment Period and Study Period 46**](#__RefHeading___Toc423700585)

[**13. Ethical Considerations 46**](#__RefHeading___Toc423700586)

[**13.1. Protection of Patients 46**](#__RefHeading___Toc423700587)

[**13.2. Informed Consent 47**](#__RefHeading___Toc423700588)

[**13.3. Protection of Privacy and Identification of Patients 47**](#__RefHeading___Toc423700589)

[**13.4. Compliance with the Protocol 47**](#__RefHeading___Toc423700590)

[**13.5. Approval by the Institutional Review Board 48**](#__RefHeading___Toc423700591)

[**13.6. Changes to the Protocol 48**](#__RefHeading___Toc423700592)

[**14. Conflict of Interest and Funding Source 48**](#__RefHeading___Toc423700593)

[**15. Publication of Study Results 48**](#__RefHeading___Toc423700594)

[**16.1. Principal Investigator 49**](#__RefHeading___Toc423700595)

[**16.2. Study Administrative Office 49**](#__RefHeading___Toc423700596)

[**16.3. Protocol Committee Members 49**](#__RefHeading___Toc423700597)

[**16.4. Central pathological Evaluation Panel Member 49**](#__RefHeading___Toc423700598)

[**16.5. Statistical Analysis/Data Management 49**](#__RefHeading___Toc423700599)

[**16.6. Data and Safety Monitoring Board 49**](#__RefHeading___Toc423700600)

[**16.7. Planned Study Sites 49**](#__RefHeading___Toc423700601)

[**Appendix 3　Package inserts 53**](#__RefHeading___Toc423700603)

**1.**  **Objective**

To evaluate the efficacy and safety of neoadjuvant chemotherapy in combination with zoledronic acid in patients with stage IIA to IIIB HER2-negative primary breast cancer

**1-1. Primary endpoint**

Pathological complete response rate: pCR*

* The definition of the NSABP B-18 study is used: pathological complete response is “the absence of invasive disease in the breast, irrespective of the presence of any residual disease in the auxiliary nodes.”

**1-2. Secondary endpoints**

1. Tumor response rate (proportion of Complete Response [CR] + Partial Response [PR])

2. Proportion of breast-conserving surgery

3. Disease free survival rate

4. Adverse events

**2. Background and Rationale for the Study**

**2.1.　 Treatment of Breast Cancer and Preoperative (Neoadjuvant) Chemotherapy**

Breast cancer forms clinically undetectable systemic micrometastasis from a relatively early stage of the disease. Extensive surgery cannot prevent micrometastasis. Local treatment including surgery and radiation therapy has been found to be insufficient to control micrometastasis, and postoperative systemic pharmacotherapy such as chemotherapy and endocrine therapy has been utilized since the1970s. Its importance is increasing in recent years.

Preoperative (neoadjuvant) chemotherapy has been examined since the late 1980s in the expectation that the prognosis will be improved by early prevention of micrometastasis that determine the prognosis. The benefits of neoadjuvant chemotherapy are (1) tumor mass reduction and downstaging can be achieved and are expected to improve by tumor reduction, resulting in possible improvement in the breast conservation rate, (2) early systemic treatment can be performed for micrometastasis lesions, (3) sensitivity to drugs can be determined early, and (4) treatment can be given prior to surgery that will desctroy the tumor vascular system before the tumors are destroyed by surgery. Improved disease free survival (DFS) and overall survival (OS), and improved cure rate are expected from the therapy. Thus, a number of clinical studies were conducted with an aim of demonstrating the benefits of neoadjuvant chemotherapy. In some studies of anthracyclines, such as doxorubicin, high response rates of 70% to 80% were reported.2,3,4)

The NSABP B-18 study, a comparison of preoperative and postoperative chemotherapy with doxorubicin/cyclophosphamide (AC 60/600), reported that there was no significant difference between pre- and postoperative chemotherapy in the influence on the prognosis.5) In Europe, the EORTC 10902 study conducted in Europe that compared preoperative and postoperative chemotherapy with fluorouracil/epirubicin/cyclophosphamide (FEC 600/60/600) also showed that there was no significant difference.

**2.2.　 Clinical Study Results of Neoadjuvant Chemotherapy**

In the NSABP B-18 study,5) mentioned above, among patients who achieved clinical complete response (cCR), improvement in prognosis was clearly shown in patients with pCR compared with those with residual invasive cancer cells, with 9-year overall survival (OS) of 85% vs. 73% (p=.0008), and DFS of 75% vs. 58% (p=.00005), respectively. In recent years, drugs by which pCR can be achieved have been explored in more patients in neoadjuvant chemotherapy.

In the NSABP B-27 study, patients were randomly assigned to receive either 4 cycles of preoperative AC followed by 4 cycles of docetaxel, or 4 cycles of preoperative AC, or 4 cycles of preoperative AC followed by postoperative 4 cycles of docetaxel. The study results showed that breast conserving rates were 63.7% and 61.6% in the AC followed by docetaxel groups and in the AC group, respectively, showing no significant difference. pCRs were 26.1% and 13.7% in the AC followed by docetaxel groups and in the AC group, respectively, showing a significant difference.7) There was no difference in DFS and OS between the 3 groups. However, results were better in patients who achieved pCR, DFS and OS compared to those who did not achieve pCR (Hazard ratio, 0.45 and 0.33, respectively). 8)

According to a report by Smith et al. from a 5-year follow-up of the Aberdeen trial of sequential dosing with 4 cycles of CVAP followed by 4 cycles of docetaxel vs. 8 cycles of CVAP,9) significant improvement was shown in the breast-conserving rate (67% vs. 48%), pCR rate (34% vs. 16%), and 5-year survival (97% vs.78%) .

M.D. Anderson Cancer Center conducted a study of sequential dosing of taxan and anthracycline where weekly paclitaxel and paclitaxel every 3 weeks are compared.10) The result showed that a high pCR rate of 29% was achieved in the weekly paclitaxel group. In patients who achieved pCR, both DFS and OS were favorable. A study that compared concurrent dosing and sequential dosing of anthracycline and taxan, the pCR rate was higher in sequential dosing than in concurrent dosing.11)

In conclusion, preoperative chemotherapy can achieve survival comparable to that achieved by postoperative chemotherapy of the same regimen in early-stage operable breast cancer. Preoperative chemotherapy improves breast-conserving rate. The sequential addition of taxan to the treatment with anthracycline further improves pCR rate. Patients who achieved pCR have favorable prognosis.

**2.3.　Zoledronic Acid**

Zoledronic acid (Zometa®) is a nitrogen-containing bisphosphonate that strongly inhibits bone resorption, discovered by Swiss-based Novartis. The bisphosphonate inhibits osteoclastic bone resorption after being internalized by osteoclasts in the body.12)

Novartis discovered that zoledronic acid had the most potent pharmacological action among bisphosphonates based on the results from preclinical studies during and after 1986.13) From September of 1993, in Europe and the U.S., they conducted clinical trials of zoledronic acid and confirmed its efficacy in patients with hypercalcemia caused by malignant tumors.14) In Japan, a bridging study was conducted in patients with hypercalcemia caused by malignant tumors to extrapolate the data from overseas studies into Japanese patients, and demonstrated efficacy.15) In October of 2004, zoledronic acid was approved for hypercalcemia caused by malignant tumors in Japan. Also, from August of 1994, clinical trials were conducted in Europe and the U.S. to evaluate efficacy in solid tumor metastasis and bone lesions in multiple myeloma, and confirmed that zoledronic acid prevents skeletal-related events (SRE).16-21)

Zoledronic acid was approved for multiple myeloma and bone metastasis in solid tumors in the U.S. in February of 2002 and in EU countries in July of 2002. In Japan, a double-blind controlled study of zoledronic acid vs. placebo was conducted in patients with bone metastases from breast cancer and the results showed that zoledronic acid significantly reduced a risk of developing SRE.22) The domestic and overseas study results were compiled into a clinical data package for approval application of zoledronic acid, and it was approved to use for the treatment of bone lesions in multiple myeloma and bone lesions in solid tumor metastasis 23)

**2.4.　 Treatment with Zoledronic Acid in Patients with Breast Cancer**

The ABCSG-12 study, a clinical trial in postoperative breast cancer patients, compared hormone therapy using a LHRH agonist plus tamoxifen or anastrozole with or without zoledronic acid (4 mg given intravenously every 6 months) in premenopausal patients. The results were reported at ASCO 2008, showing that disease free survival (DFS) was significantly improved in the group that received hormone therapy with zoledronic acid, compared with the group that received hormone therapy alone (HR 0.642, p = 0.011). The therapy with zoledronic acid also reduced the occurrence of distant metastasis compared with the hormone therapy alone group (29 vs. 41) as well as that of local recurrence (10 vs. 20).24)

In the Z-FAST and ZO-FAST studies, postmenopausal women with hormone-positive breast cancer received postoperative hormone therapy with letrozole plus either upfront or delayed-start zoledronic acid (4 mg given intravenously every 6 months). The delayed-start group started zoledronic acid if bone loss or pathological fracture occurred. The upfront group showed significant improvement in DFS in both Z-FAST and ZO-FAST studies.25)

**2.5.　 Zoledronic Acid as a Neoadjuvant Chemotherapy Drug in Patients with Breast Cancer**

Winter MC et al. presented the results of the AZURE (Adjuvant Zoledronic Acid in Breast Cancer) study, in which the use of zoledronic acid in addition to standard neoadjuvant chemotherapy was evaluated in premenopausal or postmenopausal patients with stage II/III breast cancer, at San Antonio Breast Cancer Symposium in 2008. 26)
While the diameter of pathological invasion was 42.4 mm in the chemotherapy alone group, there was a significant reduction to 28.2 mm when zoledronic acid 4 mg was added every 3 to 4 weeks for 6 doses to standard 6-month chemotherapy. In addition, the pCR rate was 5.8% in the chemotherapy alone group and 10.9%, significant improvement, in the chemotherapy plus zoledronic acid group.

**2.6.　 Mechanism of Anti-tumor Activity of Bisphosphonates**

It was reported that one of nitrogen-containing bisphosphonates, zoledronic acid, directly induces tumor cell apoptosis in vitro showing its anti-tumor activity, and the apoptosis effect was coordinately enhanced by concomitant use of anti-tumor drugs. It was also reported that it had properties to inhibit adhesion and invasion to the bone matrix, to inhibit angiogenesis, and to promote immune system functioning.27)
Furthermore, there is a possibility that bisphosphonates may reduce disseminated tumor cells (DTC) in the bone marrow, causing a reduced supply of cancer stem cells in the bone marrow, and consequently contribute to preventing recurrence.
An anti-tumor effect of a bisphosphonate has also been recently reported to activate γδT cells through increasing isopentenyl pyrophosphate. 28)

**2.7.　 Rationale for the Study**

Based on the current evidence and prior studies explained above, this study aims to demonstrate anti-tumor effects of zoledronic acid in patients with operable HER2 negative breast cancer, receiving FEC100 followed by weekly paclitaxel (or weekly paclitaxel followed by FEC100), which is a recommended neoadjuvant chemotherapy regimen, with a primary endpoint of pCR.

**3. Criteria and Definitions in the Study**

## 3.1. Clinical Stage Classification

The General Rules for Clinical and Pathological Recording of Breast Cancer (16th edition, 2008) will be used.

In conformity with UICC-TNM Classification (6th edition, 2002).

### 3.1.1T: Primary tumor ^note1^

|  | | Size (cm) | Fixation to chest wall ^note2^ | Edema or ulceration of the skin of the breast, or satellite skin nodules |
| --- | --- | --- | --- | --- |
| TX | | Cannot be assessed | | |
| Tis | | Carcinoma *in situ* with no evidence of an invasive component or Paget disease with no tumor | | |
| T0 | | No evidence of primary tumor ^note 3, 4^ | | |
| T1^note 5^ | | ≦ 2.0 | － | － |
| T2 | | 2.0 ＜  ≦ 5.0 | － | － |
| T3 | | 5.0 ＜ | － | － |
| T4 | a | Tumor of any size | ＋ | － |
|  | b |  | － | ＋ |
|  | c |  | ＋ | ＋ |
|  | d | Inflammatory carcinoma ^note 6^ | |  |

Note 1: T is comprehensively assessed by clinical examination (inspection and palpation) and imaging studies.

Note 2: Chest wall includes ribs, breastbones, intercostalis muscles, and anterior serratus muscles, but not pectoral muscles.

Note 3: Primary tumor cannot be detected by clinical examination (inspection and palpation) or imaging studies (mammography, ultrasonography).

Note 4: Cases of nipple discharge and calcification seen on mammogram are not assessed as T0. Assessment is deferred until the final pathological diagnosis determines the case as Tis, T1mic, or other.

Note 5: Subclassified into a (tumor ≤ 0.5), b (0.5 < tumor ≤ 1.0), and c (1.0 < tumor ≤ 2.0).
However, a tumor with the greatest diameter of histological invasion less than 0.1 cm is additionally described as T1mic.

Note 6: Inflammatory carcinoma usually shows no evidence of tumor, but diffuse redness of the skin, edema, and sclerosis.

Note 7: For multiple intraductal tumors, the highest classification T is used.

### 3.1.2 N: Regional Lymph Node note 1

|  | | Ipsilateral axillary lymph node | | Parasternal lymph node ^note 2^ | Ipsilateral infraclavicular lymph node | Ipsilateral superclavicular lymph node |
| --- | --- | --- | --- | --- | --- | --- |
|  |  | Movable | Fixed (to the surrounding tissue or each other) |  |  |  |
| NX | | Cannot be assessed | | | | |
| N0 | | － | － | － | － | － |
| N1 | | ＋ | － | － | － | － |
| N2 | a | － | ＋ | － | － | － |
|  | b | － | － | ＋ | － | － |
| N3 | a | ＋/－ | ＋/－ | ＋/－ | ＋ | － |
|  | b | ＋　　or　　　 ＋ | | ＋ | － | － |
|  | c | ＋/－ | ＋/－ | ＋/－ | ＋/－ | ＋ |

Note 1: Diagnosis of lymph node metastasis is made by palpation and imaging studies.

Note 2: If metastasis to the parasternal lymph nodes is unsearched, the case is handled as (−).

### 3.1.3 M: Distant metastasis

MX Cannot be assessed.

M0 No distant metastasis

M1 Distant metastasis

### 3.1.4　TNM Classification

|  | | T0 | T1 | T2 | T3 | T4 |
| --- | --- | --- | --- | --- | --- | --- |
| M0 | N0 |  | Ⅰ | IIA | IIB | IIIB |
|  | N1 | IIA | IIA | IIB | IIIA | IIIB |
|  | N2 | IIIA | IIIA | IIIA | IIIA | IIIB |
|  | N3 | IIIB | IIIB | IIIB | IIIB | IIIB |
| M1 | | IV | IV | IV | IV | IV |

Stage 0: Tis, noninvasive cancer

Patients who fall into the white boxes without shading will be included in the study.
(T1c-3, N0-1, and M0 of stages II A to III B)

SHAPE \* MERGEFORMAT ： Not applicable

Stage I to IV: Invasive cancer

## 3.2. Histological Classification

The General Rules for Clinical and Pathological Recording of Breast Cancer (16th edition, 2008) will be used.

1 Noninvasive carcinomas

1a. Noninvasive ductal carcinoma

1b. Lobular carcinoma *in situ*

2 Invasive carcinomas

2a. Invasive ductal carcinoma

2a 1 Papillotubular carcinoma

2a 2 Solid-tubular carcinoma

2a 3 Scirrhous carcinoma

2b. Special types

2b 1 Mucinous carcinoma

2b 2 Medullary carcinoma

2b 3 Invasive lobular carcinoma

2b 4 Adenoid cystic carcinoma

2b 5 Squamous cell carcinoma

2b 6 Spindle cell carcinoma

2b 7 Apocrine carcinoma

2b 8 Carcinoma with cartilaginous and/or osseous metaplasia

2b 9 Tubular carcinoma

2b 10 Secretory carcinoma (Juvenile carcinoma)

2b 11 Invasive micropapillary carcinoma

　　　　 2b 12 Matrix-producing carcinoma

2b 13 Other

3 Paget’s disease

**3.3. Assessment of Change in Tumor Burden (Criteria for Clinical Response Evaluation)**

Assessment of tumor response conforms with RECIST.

Lesions identified as target lesions will be evaluated and classified into the following 4 categories.

While clinical tumor response will be assessed based on unidimensional measurements, bidimensional measurements will be taken at examination and all measurements will be recorded.

If daughter nodules are present in the same region, the largest lesion will be chosen as the target lesion for clinical response evaluation and its diameter will be reported.

Response of the target lesions (Confirmation after 4 weeks is not required.)

1) CR: Complete Response
Disappearance of all target lesions including secondary changes due to the tumor.

2) PR: Partial Response
At least a 30% decrease in the largest diameter of target lesion, taking as reference the baseline largest diameter.

3) SD: Stable Disease
Neither sufficient shrinkage to qualify for PR nor sufficient increase to qualify for PD.

4) PD: Progressive Disease
At least a 20% increase in the largest diameter of target lesion, taking as reference the baseline largest diameter.

**3.4. Histological Response Evaluation Criteria**

　1. Evaluation Criteria

Grade 0 No response

Almost no changes are observed in cancer cells after treatment.

Grade 1 Minor response

1a） Mildly effective
Minor changes are observed in cancer cells regardless of the size of the tumor area.

　　Significant changes are observed in approximately less than one third of cancer cells.

1b）Moderately effective

　　Significant changes are observed in approximately one third or more to less than two thirds of cancer cells.

Grade 2 Major response

2a）Highly effective

　　　　Significant changes are observed in approximately two thirds or more of cancer cells, provided that viable lesions are evident.

　　2b）Extremely effective

　　　　Almost as effective as complete response (Grade 3), but a very small number of cancer cells still remain.

　　Grade 3 Complete response

　　　　　All cancer cells have undergone necrosis or disappeared, or are replaced with granuloma-like tissues or fibrotic foci.

The response evaluation criteria are summarized in the following table.

| Degree of change  Ratio of the area with changes | Mild change | Significant change |
| --- | --- | --- |
| area < 1/3 | 1a | 1a |
| ≤ 1/3 area < 2/3 | 1a | 1b |
| area ≥ 2/3 | 1a | 2 |

2. Pathological complete response: pCR

pCR in the study is defined as complete absence of invasive cancer cells in the breast, irrespective of the presence of any residual disease in the auxiliary nodes.

Note1: Mild changes are mild degenerative findings in cancer cells, i.e., changes to a degree that cancer cells are viable (including cells with acidophilic cytoplasm, vacuoles, and expanding nuclei).

Note 2: Significant changes are highly degenerative findings in cancer cells, i.e., changes to a degree that cancer cells are almost inviable or in the direction of disintegration (degeneration or necrosis).

Note 3: The ratio of the area of changes will be calculated based on the estimated tumor size from the baseline, not from the number of residual cancer cells (e.g., If a tumor of 2 cm before treatment has changed to a small amount of residual cancer cells, it will be assessed as Grade 2b, not Grade 1a.

Note 4: Biopsy specimens after treatment (needle biopsy and partially resected tumor specimens) will not be used for final assessment. Only histological findings will be recorded for each specimen.

Note 5: In the borderline cases where difficulty arises, a lower grade will be chosen.

Note 6: Assessment of Grade 3 requires examination of multiple tissue sections.

**3.5. Performance Status (PS)**

Japanese translation of ECOG scale will be used.

| Grade | Performance Status |
| --- | --- |
| 0 | Fully active, able to carry on all pre-disease performance without restriction |
| 1 | Restricted in physically strenuous activity but ambulatory and able to carry out works of a light and sedentary nature, e.g., light house work, office work |
| 2 | Ambulatory and capable of all self-care but unable to carry out any work activities. Up and about more than 50% of waking hours |
| 3 | Capable of only limited self-care, confined to bed or chair more than 50% of waking hours |
| 4 | Completely disabled. Cannot carry on any self-care. Totally confined to bed or chair |

**3.6. Definition of Postmenopausal Patients**

Postmenopausal patients in the study will be those who meet any of the following criteria.

1. Patients aged 55 years or older, and 1 or more years have passed since her last menstruation.

2. Patients with amenorrhea who are younger than 55 years (natural menopause, drug induced menopause, patients who underwent hysterectomy) should have an FSH or E_2_ level that meets menopause criteria of the study site.

3. Patients who underwent bilateral ovariectomy.

**3.7. Assessment of Recurrence**

**3.7.1 Definition and Categories of Recurrence**

(See Chapter 3.2 Recurrence, *General Rules for Clinical and Pathological Recording of Breast Cancer*, 16th edition.)

Recurrence is defined as the return of histologically (cytologically) confirmed breast cancer after treatment (surgery, radiation therapy, chemotherapy) and after a period of time during which the cancer cannot be clinically detected. Multiple cancers are excluded. Recurrence is classified as follows, depending on the site where recurrent tumors have returned.

a) Recurrence in the conserved breast

b) Local (chest wall on the affected side) recurrence
The chest wall mentioned here is an area surrounded by the following boundaries: the upper boundary of the inferior border of the clavicle, the lower boundary of the costal arch, the medial boundary of the midline of the sternum, and the lateral boundary of the anterior border of the latissimus dorsi muscle. In cases where difficulty arises in deciding due to recurrent tumor’s proximity to the boundary, it is considered to be local recurrence.

c) Regional lymph node recurrence

d) Distant recurrence

　　In addition to the categories above, the site^*1^ and the date^*2^ of recurrence will be recorded.

　　*1: For distant organs, coding will be performed similarly to M in the TNM classification.

　　*2: Defined as the date when recurrence is objectively confirmed by a physician (e.g. the date of examination).

**3.7.2 Diagnosis of Recurrence**

Criteria for definitive diagnosis of recurrence are as follows.

1) Recurrence in the conserved breast
If definitive diagnosis is established by biopsy or cytological examination, it is considered recurrence.

2) Local (chest wall on the affected side) recurrence
If definitive diagnosis is established by biopsy or cytological examination, it is considered recurrence.

3) Regional lymph node
If definitive diagnosis is established by any of biopsy, cytological examination, or imaging studies, it is considered recurrence.

4) Metastasis to distant organs

1. Contralateral breast

If definitive diagnosis is established by cytological examination or biopsy and evidence of metastasis is confirmed clinically and pathologically, it is considered recurrence. A diagnosis of metachronous bilateral breast cancer is not considered as recurrence, but as secondary cancer (multiple primary cancers).

(2) Distant lymph node
If definitive diagnosis is established by any of biopsy, cytological examination, or imaging studies, it is considered recurrence.

(3) Lung
If multiple nodular shadows consistent with lung metastasis are observed by imaging studies (such as plain chest x-ray and CT), it is considered recurrence.
If a new isolated nodule that is difficult to be differentiated from primary lung cancer appears, it will be considered recurrence when appropriate differential diagnosis is made and confirms it is metastasis.
If it is primary lung cancer, it is considered concurrent secondary cancer.

(4) Bone marrow
If a diagnosis is made definitive by bone marrow puncture, it is considered recurrence.

(5) Bone
If osteolysis or osteoplastic changes are observed on bone x-ray, or abnormalities are observed on bone scintigram or bone MRI and other conditions are ruled out, it is considered recurrence.

(6) Pleura (pleural effusion)
If cytological examination or pleural biopsy is positive, it is considered recurrence.

(7) Meninges
If a diagnosis is made definitive by cytological examination, it is considered recurrence.

(8) Ascites (peritoneum), pericardial effusion
If definitive diagnosis is established by cytological examination or biopsy, it is considered recurrence.

(9) Eyes
If definitive diagnosis is confirmed by fundus examination, CT, or MRI, it is considered recurrence.

(10) Liver
If definitive diagnosis is established by ultrasonography, CT, or MRI, it is considered recurrence.

(11) Skin

If definitive diagnosis is established by biopsy or cytological examination, it is considered recurrence.

(12) Brain, spinal cord
If definitive diagnosis is established by CT or MRI, it is considered recurrence.

(13) Other
If definitive diagnosis is established by cytological or histological examination, or if definitive diagnosis is not established by cytological or histological examination but other conditions are ruled out, it is considered recurrence.

5) Diagnosis of recurrence using tumor markers

Diagnosis of recurrence is not made based solely on tumor marker elevation.

If multiple tumor specific markers have simultaneously elevated by more than a certain percentage, or if a single tumor marker continuously rises over time, recurrence is very likely. In such cases, definitive diagnosis will be made appropriately after imaging studies.

**3.7.3 Date of Recurrence**

Among the dates of cytological examination, histological examination, and imaging studies, the earliest date when recurrence is confirmed will be the date of recurrence (decision based on the First Evidence Principle).

**4. Patient inclusion criteria**

**4.1. Inclusion criteria**

1. Women who are histologically diagnosed as having invasive breast cancer using biopsy

2. Patients with clinical stage 2A-3B and meeting one of the followings

1) Tumor measuring 3 cm or more in diameter.

2) Tumor measuring 2 cm or more in diameter and axillary lymph node metastasis (N1) is confirmed by biopsy or cytology.

3. Immunohistochemistry (IHC) score of 0 or 1+ by Hercep Test. If the score is 2+, the patients need to be proven HER2-negative by fluorescence *in situ* hybridization.

4. Patients who are ≥ 20 to ≤ 70 years of age at the time of enrollment

5. Patients with ECOG performance status of 0 or 1

6. Patients who have normal organ function as described below, in principle, within 14 days before enrollment

1) WBC ≥ 3,000 /mm^3^ and Neutrophils ≥ 1,500 /mm^3^

2) Hb ≥ 9.0 g/dL

3) Platelet counts ≥ 100,000 /mm^3^

4) AST or ALT ≤ 2.5 x upper limits of normal (ULN) defined by each study sit

5) Serum Creatinine ≤ 1.5 mg/dl

6) Normal electrocardiogram

7) Echocardiography: left ventricular ejection fraction (EF) ≥ 50%

7. Patients who have provided written informed consent by patients

**4.2. Exclusion criteria**

1. Patients with non-invasive breast cancer or invasive micropapillary carcinoma

2. Patients with inflammatory breast cancer

3. Patients with bilateral breast cancer including metachronous contralateral breast cancer

4. Patients having previously received treatment of breast cancer such as chemotherapy, hormone therapy or radiation therapy

5. Patients with active double cancer

6. Patients have a history or comorbidity of cardiac conditions described below:

(1) a history of myocardial infarction or congestive heart failure

(2) a comorbidity of ischemic cardiac disease, arrhythmias, myocardial damage or valvular disorder

7. Patients with serious complications including infectious diseases, pulmonary fibrosis, interstitial pneumonia, peripheral neuropathy, uncontrolled diabetes, and bleeding tendency

8. Patients who are pregnant or breastfeeding, or of childbearing potential

9. Patients who have dental problems including infection of the teeth or jawbone and dental trauma, or those who are scheduled for dental or jaw surgery within the next 6 weeks (e.g., extraction, implants)

10. Patients who have viral hepatitis, and those who are HBs antigen-positive or HCV antibody-positive

11. Patients who have received bisphosphonate within the past 12 months

12. Patients who are considered unsuitable by the investigators

**5. Enrollment and Allocation**

**5.1. Enrollment Procedure**

After confirming that the patient meets all of the including criteria and does not meet any of the exclusion criteria, the investigator will put all the necessary information in the patient enrollment form and fax it to the data center.

Contact information and business hours of the data center

Kohei Akazawa

Department of Medical Informatics, Niigata University Medical & Dental Hospital

　　　TEL: 025-227-2471　(Weekdays 9:00 to 16:00)

　　　FAX：025-227-0850

　　　E-mail：[akazawa@med.niigata-u.ac.jp](mailto:akazawa@med.niigata-u.ac.jp?Subject=赤澤教授へホームページより連絡)

(Closed on national holidays, Saturdays, Sundays, and year-end and new-year holidays)

Contact information regarding patient eligibility criteria

　Administrative Office

Department of Breast Surgery, Tokyo Medical University Hospital　　TEL: 03-3342-6111 (main telephone number)

Hiroshi Kaise　E-mail：[kaise3632@yahoo.co.jp](mailto:kaise3632@yahoo.co.jp)

Kimito Yamada　E-mail：[toya0@tokyo-med.ac.jp](mailto:toya0@tokyo-med.ac.jp)

**5.2. Considerations at Enrollment**

1. A patient will not be enrolled after the start of the study treatment.

2. When the information in the enrollment form is insufficient, enrollment will not be accepted until all the required information is entered.

3. A patient enrollment number for each subject will be issued after the eligibility is confirmed at the data center.

4. Upon completion of patient enrollment, a patient enrollment confirmation will be sent by fax from the data center to the investigator. The investigator should retain the faxed copy.

5. Once a patient is enrolled, the enrollment cannot be canceled.

6. Mistakes or duplicates in enrollment, if any, should be immediately reported to the data center.

**5.3. Randomization and Allocation Adjustment Factors**

Patients will be randomly assigned to either A: chemotherapy + Zoledronic acid (CTZ group) or B: chemotherapy alone (CT group) by the data center at enrollment.

At randomization, a minimization method will be used with adjustment factors (1) lymph node metastasis* (present or absent), (2) ER expression status (positive cells ≥ 10% or < 10%), and (3) menopausal status (premenopausal or postmenopausal). Patients will be assigned to each group so that there is no uneven distribution of patients between the groups.

*Determined by imaging studies

**6. Treatment Plan and Criteria for Changes to Treatment**

**6.1. Study Treatment**

**6.1.1. . Neoadjuvant chemotherapy**

A regimen of 5-FU 500 mg/m^2^, epirubicin 100 mg/m^2^ and CPA 500 mg/m^2^ (FEC 100) will be administered on Day 1 and every 3 weeks thereafter for 4 cycles in total followed by paclitaxel 80 mg/m^2^ once weekly for 12 cycles.

A regimen of paclitaxel 80 mg/m^2^ once weekly for 12 cycles followed by FEC 100 every 3 weeks for 4 cycles is permitted. However, neoadjuvant chemotherapy regimen should be identical at each study site.

**6.1.2. Zoledronic acid**

Zoledronic acid 4 mg will be diluted in 100 mL of saline or 5% glucose injection solution, and administered intravenously over at least 15 minutes.

Zoledronic acid will be administered 7 times at an interval of 3 to 4 weeks.

The interval of 3 to 4 weeks means that one-week window of time per visit is allowed considering time lag of patient’s visit. A minimum interval of 3 weeks must be observed between each treatment session.

If zoledronic acid is administered on the same day as chemotherapy drugs, it will be administered after chemotherapy drugs.

In patients with concurrent mild renal dysfunction, creatinine clearance (CrCl) will be calculated using the following formula for dose modification.

　CrCl = (140 −age) × Body weight (kg) × 0.85/72 × Serum creatinine (mg/dL)

| CrCl value before administration (mL/min) | Recommended dose of zoledronic acid |
| --- | --- |
| ＞60 | 4.0mg |
| 50-60 | 3.5mg |
| 40-49 | 3.3mg |
| 30-39 | 3.0mg |

**6.2. Criteria for Study Treatment Discontinuation/Completion**

**6.2.1. Definition of Study Treatment Completion**

Once patients in the CT group have finished the planned neoadjuvant chemotherapy (4 cycles of FEC 100 and 12 cycles of weekly paclitaxel), the study treatment is considered completed.

**6.2.2.Criteria for Study Treatment Discontinuation**

The study treatment will be discontinued if any of the following criteria applies.

1. Evident clinical progression of breast cancer occurs and tumor response evaluation turns out to be PD.

2. The study treatment cannot be continued due to adverse events.

(1) Grade 4 nonhematological toxicity occurs.

(2)The start of a following cycle of chemotherapy is delayed 3 weeks or more due to adverse events.

(3) Any of the criteria for study treatment discontinuation in 6.3 Criteria for Modification of Study Treatment applies.

(4) Allergic reaction/hypersensitivity of Grade 3 or greater occurs during the course of weekly paclitaxel.

3. The patient wishes to discontinue the study treatment by withdrawing consent.

4. Osteonecrosis of the jaw occurs and discontinuation of zoledronic acid is considered appropriate.

5. Oral surgical procedure such as tooth extraction is required during the study period.

6. The patient is found to be ineligible after the start of treatment.

7. The patient wishes to discontinue the study.

8. Other cases where the investigator considers it difficult to continue the study treatment.

**6.3. Criteria for Modification of Study Treatment**

Dosing may be modified according to the severity of adverse events and recovery of the patient as follows.

**6.3.1. Criteria for Administration of FEC after the Second Cycle**

At the time of administration from the second cycle of FEC, the investigator should confirm that the following criteria are met before planned administration. If the criteria are not met, the patient will be followed up at an interval of 1 to 2 weeks. If all of the criteria are met, the following cycle of treatment can be started.

　(1) Hematological toxicity

　　1)　 Neutrophil counts ≥ 1,500/mm^3^

　　2)　Platelet counts ≥ 100,000/mm^3^

　　　If G-CSF is administered, the patient will be followed up for more than 3 days to confirm that this criterion is met.

　(2) Peripheral neuropathy and nonhematological toxicity other than edema: Grade 1 or less

　(3) Peripheral neuropathy and edema: Grade 2 or less

**6.3.2. 2 Criteria for Dose Reduction in FEC after the Second Cycle**

Epirubicin will be reduced by 25% if any of the following adverse events occurs during the previous cycle.

　Epirubicin:100 mg/mm^2^　→　75 mg/mm^2^

(1) Febrile neutropenia

　　Confirmed with a neutrophil count < 1,000/mm^3^ and axillary temperature of 38℃ (sublingual temperature of 38.5℃) or more. If a fever of 38.5℃ or more occurs during a period from 4 to 18 days after chemotherapy treatment, it is considered as Grade 3 or greater febrile neutropenia depending on the timing and severity of the fever. Measurement of neutrophil counts is not mandatory.

　(2)Grade 3 or greater nonhematological toxicity

　If the criterion for dose reduction is still met with a reduced dosing of epirubicin, the study treatment will be discontinued. Once the dose is reduced, it will not be increased.

**6.3.3. Criteria for the Start/Delay of Weekly Paclitaxel**

If all of the criteria for the start of weekly paclitaxel specified below are not met on the day of administration or 1 day before administration for cycles 1, 5 and 9, the administration will be delayed. Although lab tests for other cycles are not mandatory, if lab tests on the day of or 1 day before administration are performed and the criteria below are not met, the administration will be delayed.

　After the delay, if the criteria for the start of weekly paclitaxel are met within 16 days, paclitaxel will be administered. If the criteria for the start of weekly paclitaxel are not met after 16 days or more, the study treatment will be discontinued.

Criteria for the start of weekly paclitaxel

　(1) Neutrophil counts ≥ 1,000/mm3

　(2) Platelet counts ≥ 75,000/mm3

　(3) All of the peripheral neuropathy and nonhematological toxicity other than edema that occurred during the previous cycle have resolved to Grade 1 or less.

**6.3.4. Criteria for Dose Reduction of Weekly Paclitaxel**

If any of the following adverse events occurs during the previous cycle, the dose of paclitaxel will be reduced by 25%.

　Paclitaxel：80mg/mm^2^　→　60mg/mm^2^

(1) Febrile neutropenia

　(2)Grade 3 or greater nonhematological toxicity

　If the criterion for dose reduction is still met with a reduced dosing of paclitaxel, the study treatment will be discontinued. If once the dose is reduced, it will not be increased.

**6.4. Concomitant Therapy, Supportive Therapy**

**6.4.1. Prohibited Therapies**

The following therapies that may influence evaluation for the study will be prohibited during the period of study drug administration.

　(1) Other anticancer drugs

　(2) Hormone therapy (including SERMs such as raloxifene)

　(3) BRMs

　(4) Other bisphosphonates

　(5) Radiation therapy

　(6)Surgery

**6.4.2. Pretreatment Prior To Paclitaxel**

The following premedications will be administered, in principle, at least 30 minutes before paclitaxel administration.

　(1) Injection of dexamethasone sodium phosphate (dexamethasone 8 mg)

　(2) Injection of ranitidine hydrochloride (ranitidine 50 mg) or famotidine (famotidine 20 mg)

　(3) Tablets of diphenhydramine hydrochloride (diphenhydramine hydrochloride 50 mg orally administered)

**6.4.3. Permitted Concomitant Therapies**

(1) Treatment for comorbidities

　　Therapies for comorbidities that predate the study, analgesics, and over-the-counter drugs/therapies will be permitted.

　(2) Calcium and vitamin supplements

　The patient may take a calcium supplement of 500 to 610 mg and a multi-vitamin tablet (containing vitamin D 400 to 800 IU) once daily after meal during the period of study drug administration if she wishes. The choice of calcium supplement and multivitamin tablet will be at the discretion of the investigator.

　(3) Symptomatic therapy for leukopenia and neutropenia

　　A G-CSF agent should be administered in accordance with the instructions in the package insert. If the patient has a fever of Grade 4 or 38℃ or more and neutropenia of Grade 3 or more, G-CSF may be administered at the discretion of the investigator.

　(4) Symptomatic therapy for hypocalcemia

　　If a patient receiving zoledronic acid experiences hypocalcemia, calcium preparation (e.g., Calcicol® for injection) will be administered.

**6.5. Surgery**

Appropriate surgery, e.g., breast conserving surgery or mastectomy, will be performed according to the size and extent of the tumor confirmed by diagnostic imaging after the last dose of neoadjuvant chemotherapy.

**6.6. Postoperative Therapy**

Postoperative radiation therapy is optional at each study site.

Appropriate treatment will be provided taking into account of expression status of hormone receptors.

Letrozole will be used for hormone therapy in menopausal hormone-sensitive patients in principle.

**6.7. Follow-up after Surgery**

After surgery, patients will be followed up for recurrence once a year up to 5 years after enrollment.

**7.　 Potential Adverse Reactions**

**7.1.　 Potential Adverse Reactions for Each Drug**

**7.1.1. Fluorouracil(5-FU)**

**7.1.2. Epirubicin(EPI)**

**7.1.3. Cyclophosphamide(CPA)**

**7.1.4. Paclitaxel(PTX)**

**7.1.5. Zoledronic acid**

　For potential adverse reactions for each drug, see the package inserts in the Appendix.

**7.2. Assessment of Adverse Events/Adverse Reactions**

The Japanese Common Terminology Criteria for Adverse Events ver.3.0 by JCOG/JSCO (Japanese translation of NCI-Common Terminology Criteria for Adverse Events [CTCAE] v3.0) will be used for the assessment of adverse events/adverse reactions.

Adverse events data will be collected in accordance with the following policies.

(1) All adverse events that occur within 30 days from the last dose of the study drugs will be collected regardless of causes.

(2) Adverse events on and after 31 days from the last dose of the study drugs will be collected only if they are considered related to the study treatment.

**8. Endpoints, Laboratory Examinations, Evaluation Schedule**

**8.1. Screening Tests Prior To Enrollment**

The following screening tests will be performed prior to patient enrollment.

Laboratory tests will be performed within 4 weeks prior to enrollment, and imaging studies within 8 weeks prior to enrollment, in principle.

1) Patient characteristics: birth date (age), height, weight, major medical history, comorbidities, and treatment for comorbidities

2) General performance status: ECOG PS

3) Menopausal status

In patients younger than 55 years other than those who underwent bilateral ovariectomy, FSH and E_2_ will be measured to determine menopausal status.

4) Diagnosis of invasive breast cancer

Needle biopsy will be performed to diagnose invasive breast cancer,

5) Clinical stage

6) Tumor characteristics: histopathological classification. Hormone receptors (ER/PgR), HER 2 expression

ER/PgR expression status will be determined by immunohistochemistry (IHC). A result of ER/PgR expression-positive requires 10% or more positive cells in this study.

If a result of HER2 2+ is obtained from IHC, HER2 negative should be confirmed by FISH.

7) Measurement of tumor diameter: the following ① and ② are mandatory, and bidimensional measurements will be obtained.

(1) Clinical examination (inspection and palpation) or ultrasonography

(2) MRI or CT

8) Screening of the bones, liver, and lungs (Absence of metastatic lesions should be confirmed.)

(1) Bone scintigraphy, bone x-ray, or CT/MRI

(2) Chest x-ray or chest CT

(3) Abdominal CT or abdominal ultrasonography

9) Axillary lymph node metastasis: clinical examination (inspection and palpation) ± imaging studies

Sentinel lymph node biopsy will be performed before neoadjuvant chemotherapy. If a patient is considered as lymph node metastasis-negative, axillary lymph node dissection may be omitted in surgery. The method of sentinel lymph node biopsy is not specified.

10) Planned surgical procedure

11) Hematology: white blood cell counts, neutrophil counts (band leukocytes + segmented cells), hemoglobin, platelet counts

12) Blood biochemistry: AST, ALT, total bilirubin, creatinine, albumin, calcium

13) 12-lead electrocardiography at rest

14) Echocardiography

**8.2. Tests and Evaluation During Study Treatment Period**

1) General findings

PS and weight will be measured in a medical interview at patient’s visit.

2) Tumor diameter

(1)Clinical examination (inspection and palpation) or ultrasonography: bidimensional measurements

Time point: Weeks 6, 12, 16, 20, and 24

(2) MRI or CT: bidimensional measurements

　Time point: Weeks 12 and 24

3) Tumor response evaluation

　　A lesion with the largest diameter on MRI or CT images will be chosen as the target lesion. The other lesions will not be evaluated.

　　Tumor response evaluation of the target lesion will be performed by clinical examination (inspection and palpation) and MRI or CT in accordance with the RECIST criteria (3.3. Tumor Response Evaluation [Clinical Response Evaluation Criteria]).

　　If progression of the tumor becomes evident during the period of study treatment with assessment of PD, the study treatment will be discontinued. Then, appropriate surgery will be performed.

4) Study treatment, prohibited concomitant therapy

5) Hematology: white blood cell counts, neutrophil counts (band leukocyte + segmented cells), hemoglobin, platelet counts

　Time point: Prior to each treatment with FEC (the day before administration or on the day of administration)

　　　　　　Before the start of cycles 1,5, and 9 of weekly paclitaxel (the day before administration or on the day of administration)

　　　　　　At the end of the study treatment

6) Blood biochemistry: AST, ALT, total bilirubin, creatinine, albumin, and calcium

Time point: Prior to the start of zoledronic acid administration (the day before administration or on the day of administration), at the end of the study treatment

7) Adverse events

**8.3. Tests and Evaluation After Completion of Study Treatment**

1) Actual surgical procedures

　　After completion of the study treatment, the extent of surgical resection will be recorded.

　　(1) Breast-conserving surgery: code for the area affected by invasive procedure

　　(2) Mastectomy: code for the area affected by invasive procedure

　　(3) No surgical treatment (Rationale for no surgical treatment will be recorded.)

2) Tumor tissues

　　HE-stained pathological specimens will be prepared from samples collected at surgery.

　　If no surgical treatment is given, HE-stained pathological specimens will be prepared from samples collected from needle biopsy.

3) Tumor response evaluation

　　Tumor response evaluation of the target lesion will be performed by clinical examination (inspection and palpation) and MRI or CT in accordance with the RECIST criteria (3.3. Tumor Response Evaluation [Clinical Response Evaluation Criteria]).

　　Tumor response evaluation will be performed during a period between completion of neoadjuvant chemotherapy and surgical treatment.

4) Histological response evaluation

　　Central pathological evaluation will be performed using HE-stained pathological specimens collected at surgery.

8.4.　 Study Schedule

| Visit | 1 | 2 | 3 | 4 | 5 | 6 | 7-9 | 10 | 11-13 | 14 | 15-17 | 18 | 19 |
| --- | --- | --- | --- | --- | --- | --- | --- | --- | --- | --- | --- | --- | --- |
| Time (Week)  Events | Day -28-0 | Day1 | 3 | 6 | 9 | 12 | 13-15 | 16 | 17-19 | 20 | 21-23 | 24 | 28 |
| Informed consent | ○ |  |  |  |  |  |  |  |  |  |  |  |  |
| Patient enrollment | ○ |  |  |  |  |  |  |  |  |  |  |  |  |
| Patient characteristics | ○ |  |  |  |  |  |  |  |  |  |  |  |  |
| Needle biopsy | ○ |  |  |  |  |  |  |  |  |  |  |  |  |
| Tumor characteristics | ○ |  |  |  |  |  |  |  |  |  |  |  |  |
| Palpation or ultrasonography | ○ |  |  | ○ |  | ○ |  | ○ |  | ○ |  | ○ |  |
| MRI or CT | ○ |  |  |  |  | ○ |  |  |  |  |  | ○ |  |
| Bone scintigraphy or bone x-ray or MRI/CT | ○ |  |  |  |  |  |  |  |  |  |  |  |  |
| Chest x-ray/chest CT | ○ |  |  |  |  |  |  |  |  |  |  |  |  |
| Abdominal CT/abdominal echo | ○ |  |  |  |  |  |  |  |  |  |  |  |  |
| Hematology | ○ |  | ○ | ○ | ○ | ○ | (○) | ○ | (○) | ○ | (○) | ○ |  |
| Blood biochemistry | ○ |  | ○ | ○ | ○ | ○ |  | ○ |  | ○ |  | ○ |  |
| Electrocardiography | ○ |  |  |  |  |  |  |  |  |  |  |  |  |
| Echocardiography | ○ |  |  |  |  |  |  |  |  |  |  |  |  |
| FEC100 |  | ○ | ○ | ○ | ○ |  |  |  |  |  |  |  |  |
| Paclitaxel |  |  |  |  |  | ○ | ○ | ○ | ○ | ○ | ○ |  |  |
| Zoledronic acid^＊1^ |  | ○ | ○ | ○ | ○ | ○ |  | ○ |  | (○) |  |  |  |
| Adverse events |  | ○ | ○ | ○ | ○ | ○ | ○ | ○ | ○ | ○ | ○ | ○ | ○ |

*1: Only in the chemotherapy + zoledronic acid (CTZ) group

**9. Data Collection**

**9.1.　Case Report Form (CRF)**

**9.1.1 Types of CRF**

1) Patient Enrollment Form

2) Case Report

3) Treatment Completion Report

4) Pathology Report

**9.1.2　Submission of CRFs**

・Patient enrollment forms and all other CRFs will be submitted to the data center by postal mail.

　・To minimize a risk of leakage of patients’ private information, patient identification numbers must be used instead of medical record numbers of the study site for communications with the data center, such as a request for CRFs.

**10. Reporting of Adverse Events**

If a serious or unexpected adverse event described in the following section occurs, the site principal investigator will report the event to the study administrative office/chief investigator of the entire study. The latest version of the reporting form of each site will be used.

Adverse event reports shall be appropriately submitted by each site under the responsibility of the site principal investigator, in accordance with their own rules as a medical institution, to the head of each medical institution, spontaneously to the Ministry of the Health, Labour and Welfare under the reporting system of safety information on drugs etc., and spontaneously to the pharmaceutical company under the adverse event reporting system based on the Pharmaceutical Affairs Law.

**10.1. Adverse Events Requiring Reporting**

**10.1.1 Adverse Events Requiring Expedited Reporting**

(1) All deaths during the study treatment period or within 30 days of the last day of the study treatment

　　Regardless of causal relationship with the study treatment. Adverse events experienced by patients who discontinued the study treatment but have already been initiated with another treatment following the discontinuation should be reported if the events occurred within 30 days of the last day of the study treatment.

(The last day of the study treatment is considered Day 0. 30 days is counted from the following day).

(2) Unexpected Grade-4 Adverse Events

　Unexpected adverse events are any event that is not described in the package insert of the drug.

(3)Bisphosphonate Related Osteonecrosis of the Jaw (BRONJ)

Definition of Bisphosphonate Related Osteonecrosis of the Jaw (BRONJ)

　The jawbone is in the state of necrosis. Although there is no clear definition of osteonecrosis of the jaw, there is a temporary definition. If an adverse event fulfills all of the following criteria, the event is considered as osteonecrosis of the jaw.

　1) Exposed bone in the maxillofacial area that appears spontaneously or after dental surgery and persists for more than 3 to 6 weeks despite appropriate treatment

　2) Irrespective of causal relationship with infection.

　3) Irrespective of the presence of pain.

　4) No history of radiation therapy to the head or neck before the occurrence.

　5) No tumor invasion in the area.

**10.1.2 Adverse Events Requiring Normal Reporting**

　(1)Deaths on Day 31 or after the last day of the study treatment, for which a causal relationship to the study treatment cannot be ruled out.

　　Deaths suspected of being treatment-related deaths. Deaths due to the primary disease are excluded.

　(2)Potential Grade-4 adverse events

　　Potential adverse events are those described in the package insert of the drug.

　(3)Unexpected Grade-3 adverse events.

　　Grade-3 adverse events that are not described as potential adverse events in the package insert of the drug.

**10.2. Reporting Requirements and Procedures for Site Principal Investigator**

**10.2.1 Expedited Reporting**

If an adverse event to be reported in an expedited manner, the investigator should promptly report it to the site principal investigator and the head of the medical institution, and also make an oral report to the study administrative office within 24 hours. In addition, the site principal investigator will prepare a report including detailed information and send it to the study administrative office by postal mail or by fax within 15 days of knowledge of the event.

　　　The study administrative office will report the event reported in an expedited manner to the independent monitoring board to determine whether the study should be continued.

**10.2.2 Normal Reporting**

The site principal investigator will prepare a report (hospital form or any A4-size sheet) on the safety information and send it to the study administrative office by postal mail or by fax.

**11. Response Evaluation and Definition of Endpoints**

**11.1. Pathological complete response rate (pCR)**^＊^

Members of the central pathological evaluation panel will evaluate histological response in accordance with the criteria for histological evaluation (section 3.4 on page 20) to calculate the pCR rate.

* The definition of the NSABP B-18 study is used: pathological complete response is “the absence of invasive disease in the breast, irrespective of the presence of any residual disease in the auxiliary nodes.”

**11.2. Evaluation of Tumor Response**

　 Target lesions evaluable with MRI or CT will be evaluated based on the Response Evaluation Criteria In Solid Tumor (RECIST criteria, section 3.3 on page 19) to calculate the CR and PR rates.

**11.3. Proportion of Breast-conserving Surgery**

Proportion of breast-conserving surgery performed after completion of the study treatment will be calculated.

**11.4. Disease free survival**

(1）Definition

　　Time from the patient enrollment date to the occurrence of the first event.

(2）Event

　　Occurrence of any of the following is defined as an event.

1) Diagnosis of recurrence in the conserved breast, local (chest wall on the affected side) recurrence, recurrence in regional lymph node(s), or metastasis to the distant organ(s)

2) Diagnosis of metachronous breast cancer, secondary cancer (excluding cutaneous basal cell cancer, squamous cell cancer, or intraepithelial carcinoma of the uterus)

3) All deaths (irrespective of causes)

(3) Censored case

1) The last day when absence of the events above is confirmed.

2) In survival cases, the last day when survival of the patients is confirmed.

**11.5. Evaluation of Adverse Events**

　　Adverse events will be evaluated for abnormal findings, symptoms, test results, and severity in accordance with the Japanese CTCAE ver.3.0 by JCOG/JSCO, and describe them in the case report form.

**12. Statistical Considerations**

**12.1. Analysis Set**

**12.1.1. Analysis Set for Primary Endpoint and Secondary Endpoint**

Statistical analysis of the primary and secondary endpoints will be performed on a group of patients randomized and with analyzable data. Analysis on a per-protocol set (a group of patients who completed treatment stipulated in the protocol to a certain extent) will also be performed. The rate of treatment completion shall be in accordance with the criteria separately established by the study group.

**12.1.2. Safety Analysis Set**

Safety analysis will be performed on a group of patients who received at least 1 cycle of treatment in the zoledronic acid combination (CTZ) and chemotherapy alone (CT) groups.

**12.2. Statistical Analysis Plan**

Primary analysis of the study will be performed in the following procedure.

**12.2.1. Significance Test for pCR Rate**

Statistical hypothesis to be used is as follows.

　　　　　Null hypothesis: pCR rate in the CTZ group = pCR rate in the CT group

　　　　　Alternative hypothesis: pCR rate in the CTZ group > pCR rate in the CT group

　　This hypothesis will be tested with chi-square test at a one-sided significance level of 5%. Yates' correction for continuity will be used.

**12.2.2. Significance Test for Tumor Response and Proportion of Breast-conserving Surgery**

Analysis will be performed in the same manner as described in section 12.2.1.

**12.2.3. Significance Test for Disease Free Survival**

　　　Statistical hypothesis to be used is as follows.

hypothesis: 5-year survival curve in the CTZ group = 5-year survival curve in the CT group

Alternative hypothesis: 5-year survival curve in the CTZ group > 5-year survival curve in the CT group

　　　This hypothesis will be tested with stratified log-rank test at a one-sided significance level of 5%. Stratification factors are the following three factors: lymph node metastasis, ER expression status, and menopausal status. The odds ratio of CTZ group to the CT group for each background variable will be estimated using a Cox proportional hazard model.

**12.3. Planned Sample Size, Enrollment Period and Study Period**

(1) Planned sample size: 180 patients (90 patients in one group)

Rationale for sample size calculation

The pathological complete response (pCR) rate was 25% in a study of neoadjuvant chemotherapy in patients with primary breast cancer conducted by Toi *et al*.^1)^

While the pCR rate of neoadjuvant chemotherapy in AZURE study was 5.8% in the chemotherapy alone group, it was 10.9% in the zoledronic acid combination group, approximately 1.9-fold. Similarly, if the pCR rate of FEC100 followed by weekly paclitaxel in combination with zoledronic acid is 1.9-fold of that of chemotherapy alone in this study, it will be 47.5%.

Therefore, assuming that the pCR rate is 25% in the chemotherapy alone (CT) group and 45% in the zoledronic acid combination (CTZ) group, a sample size of 80 patients in one group will be necessary to achieve the power of 80% with a one-sided significance level of 5%.

A total of 180 patients will be enrolled in both groups, with potential dropouts and withdrawals.

(2) Study Period

　　 Enrollment period: from March 2010 through June 2012

Study period: from January 2010 through December 2017, for 5 years after enrollment

Revision of the protocol is not required for an extension of the enrollment period of less than within 6 months.

**13. Ethical Considerations**

**13.1. Protection of Patients**

All researchers involved in this study will conduct the study in accordance with the Declaration of Helsinki and the Ethical Guideline for Clinical Research (Public Notice No. 415, 2008, issued by the Ministry of Health, Labour and Welfare).

**13.2. Informed Consent**

The study investigator will provide an information form approved by the institutional review board to the patient before enrollment to explain the following, and obtain voluntary written informed consent to participate in the study from the patient.

Items to be explained

1. This is a clinical trial.
2. This is not a study of a new drug.
3. The purpose of the study
4. Patients to be included in the study
5. Methods of the study
6. Benefits of the study treatment
7. Potential side effects
8. Other treatment options
9. Expected benefits and potential drawbacks of participating in the study
10. Compensation for health damage
11. Payment
12. Refusal and withdrawal of consent
13. Protection of privacy
14. Instructions to follow during the study
15. Contact information and consulting desk for the study
16. Freedom to ask questions

**13.3. Protection of Privacy and Identification of Patients**

In this study, since tumor samples collected during the time of surgical treatment will be used, researchers other than the surgeon who collects the samples will not have direct contact with patients. Personal information including clinical and pathological information will be anonymized in a linkable fashion, and samples will be controlled with 2-digit serial numbers. Unstained specimens with control numbers will be used as pathological specimens in this study.

All information will be destroyed in an irreproducible fashion after the study is completed. If the information is to be used for similar studies, approval from the Institutional Review Board will be obtained first.

**13.4. Compliance with the Protocol**

Researchers participating in the study will conduct the study in compliance with the study protocol as far as the safety and human rights of the patients are protected.

**13.5. Approval by the Institutional Review Board**

The study protocol, informed consent and assent forms for patients, and the appropriateness of the study must be reviewed and approved by the Institutional Review Board/Research Ethics Board prior to the study.

**13.6. Changes to the Protocol**

If any need of making an amendment to the protocol arises during the study, the principal investigator will discuss with the Data and Safety Monitoring Board as necessary and decide what amendment should be made. The principal investigator will promptly notify the study investigators of the contents of and the reason for the changes in writing.

　　If any modifications to the protocol may significantly impact the conduct of the study, the principal investigator will report to the heads of the medical institutions for approval for the changes and also obtain approval by the Institutional Review Board/Research Ethics Board.

**14. Conflict of Interest and Funding Source**

There are no conflicts of interest in the plan, conduct, and publication of the study. The study will be financed from research funds raised by the participating institutions themselves.

**15. Publication of Study Results**

Study results will be presented at a relevant academic meeting and published as a research paper.

　　The names and the order of the authors in the academic presentation and research paper will be approved by the principle investigator and the study administration office before publication.

**16. Study Organization**

**16.1. Principal Investigator**

Norio Kohno

Department of Breast Oncology, Tokyo Medical University Hospital

**16.2. Study Administrative Office**

Hiroshi Kaise

Department of Breast Oncology, Tokyo Medical University Hospital

**16.3. Protocol Committee Members**

Shintaro Takao

Department of Breast Surgery, Hyogo Cancer Center

Masaru Miyashita

Department of Surgery, Konan Hospital

Yoshie Hasegawa

Department of Breast Surgery, Hirosaki Municipal Hospital

Hirokazu Tanino

Department of Breast Surgery, Naga Hospital

**16.4. Central pathological Evaluation Panel Member**

Takeshi Sasaki

Department of Pathology, Yokohama City University Medical Center

**16.5. Statistical Analysis/Data Management**

Kohei Akazawa

Department of Medical Informatics, Niigata University Medical & Dental Hospital

**16.6. Data and Safety Monitoring Board**

Nobuaki Sato

Department of Breast Surgery, Niigata Cancer Center Hospital

Toshio Tabei

Department of Breast Oncology, Saitama Cancer Center

Hiromitsu Jinno

Department of General and Gastrointestinal Surgery, Keio University Hospital

**16.7. Planned Study Sites**

　Appendix 1

**17. References**

1) Toi M, Nakamura S, Kuroi K, et al: Phase 2 study of preoperative sequential FEC and docetaxel predicts of pathological response and disease free survival. *Breast Cancer Res Treat* 110: 531-539, 2008

2) Fisher B, et al: Effect of preoperative chemotherapy on the outcome of women with operable breast cancer. *J Clin Oncol* 16: 2672-2685, 1998

3) Bonadonna G, et al: Primary chemotherapy in operable breast cancer: Eight-year experience at the Milan Cancer Institute. *J Clin Oncol* 16: 93-100, 1998

4) Feldmann L, Hortobagyi G: Pathological assessment of response to induction chemotherapy in breast cancer. *Cancer Res* 46: 2578-2581, 1986

5) Wolmark N, et al : Prospective chemotherapy in patients with operablr breast cancer: Nine-year results from National Surgical Adjuvant Breast and Bowel Project B-18. *J Natl Cancer Inst Monogr* 30: 96-102, 2001

6) van der Hage JA, et al: Preoperative chemotherapy in primary operable breast cancer: Results from the European Organization for Research and Treatment of Cancer Trial 10902. *J Clin Oncol* 19: 4224-4237, 2001

7) Smith, et al: Neoadjuvant Chemotherapy in Breast Cancer: Significantly Enhanced Response With Docetaxel . *J Clin Oncol* 20: 1456-1466, 2002

8) Bear HD, Anderson S, Brown A, et al: The Effect on Tumor Response of Adding Sequential Preoperative Docetaxel to Preoperative Doxorubicin and Cyclophosphamide: Preliminary Results From National Surgical Adjuvant Breast and Bowel Project Protocol B-27. *J Clin Oncol* 21: 4165-4174, 2003

9) Bear HD, Anderson S, Smith RE, et al: Sequential preoperative or postoperative docetaxel added to preoperative doxorubicin plus cyclophosphamide for operable breast cancer:National Surgical Adjuvant Breast and Bowel Project Protocol B-27. *J Clin Oncol* 24: 2019-2027, 2006

10) Green MC, Buzdar AU, Smith T, et al : Weekly paclitaxel improve pathologic complete remission in operable breast cancer when compared with paclitaxel once every 3 weeks. *J Clin Oncol* 23: 5983-5992, 2005

11) von Minvkwitz G, Raab G, Caputo A, et al: Doxorubicin with cyclophosphamide followed by docetaxel every 21 days compared with doxorubicin and docetaxel every 14 days as preoperative treatment in operable breast cancer. *J Clin Oncol* 23: 2675-2685, 2005

12) Coxon FP, Luckman SP, van’t Hof R, et al: Geranygeranylated proteins regulate osteoclast formation, morphology, function, and survival: Inhibition of prenylations by bisphosphonates and GGTI-298. *J Bone Miner Res* 15, 1467-1476, 2000

13) Green JR, Muller K, Jaeggi KA: Preclinical pharmacology of CGP42446, a new potent heterocyclic bisphosphonate compound. *J Bone Miner Res* 9, 745-751, 1994

14) Major P, Lortholary A, Hon J, et al: Zoledronic acid is superior to pamidronate in the treatment of hypercalcemia of malignancy: A pooled analysis of two randomized, controlled clinical trials. *J Clin Oncol* 19, 558-567, 2001

15) Kawada K, Minami H, Okabe K, et al: A multicenter and Open Label Clinical trial of Zoledronic Acid 4mg in Patients with Hypercalcemia of Malignancy. *Jpn J Clin Oncol* 35, 28-33, 2005

16) Rosen LS, Gordon D, Antonio S, et al: Zoledronic acid versus pamidronate in the treatment of skeletal metastasis in patients with breast cancer or osteolytic lesions of multiple myeloma: a PhaseⅢ, double blind, comparative trial. *Cancer J* 7, 377-387, 2001

17) Rosen LS, Gordon D, Kaminski M, et al: Long-term efficacy and safety of zoledronic acid compared with pamidronate disodium in the treatment of skeletal complications in patients with advanced multiple myeloma or breast carcinoma: A randomized, double-blind, multicenter, comparative trial. *Cancer* 98, 1735-1744, 2003

18) Rosen LS, Gordon D, Tchekmedyian S, et al: Zoledronic acid versus placebo in the treatment of skeletal metastases in patients with lung cancer and other solid tumors: A phase Ⅲ, double-blind, randomized trial – The zoledronic acid lung cancer and other solid tumors study group. *J Clin Oncol* 21, 3150-3157, 2003

19) Rosen LS, Gordon D, Tchekmedyian S, et al: Long-term efficacy and safety of zoledronic acid in the treatment of skeletal metastases in patients with nonsmall cell lung carcinoma and other solid tumors: A randomized, phase Ⅲ, double-blind, placebo-controlled trial. *Cancer* 100, 2613-2621, 2004

20) Saad F, Gleason DM, Murray R, et al: A randomized, placebo-controlled trial of zoledronic acid in patients with hormone-refractory metastic prostate carcinoma. *J Natl Cancer Inst* 94, 1458-1468, 2002

21) Saad F, Gleason DM, Murray R, et al: Long-term Efficacy of Zoledronic Acid for the Prevention of Skeletal Complications in Patients With Metastatic Hormone-Refractory Prostate Cancer. *J Natl Cancer Inst* 96, 879-882, 2004

22) Kohno N, Aogi K, Minami H, et al: Zoledronic Acid Significantly Reduces Skeletal Complications Compared With Placebo in Japanese Women With Bone Metastases From Breast Cancer: A Randomized, Placebo-Controlled Trial. *J Clin Oncol* 23, 3314-3321, 2005

23) Novartis Pharma K.K., Medicine Interview Form for Zometa® 4 mg Injection, January 2007 (5^th^ version of new form)

24) Gnant M, Mlineritsch B, Schippinger W, *et al:* Endocrine Therapy plus Zoledronic Acid in Premenopausal Breast Cancer. *N Eng J Med.* 360, 679-91, 2009

25) Winter MC, Thorpe HC, Burkinshaw R, et al: The addition of zoledronic acid to neoadjuvant chemotherapy may influence pathological response – exploratory evidence for direct anti-tumor activity in breast cancer. Poster session #5101, 2008, SABCS

26) Eidtman H, Bundred NJ, Deboer R, et al: The effect of zoledronic acid on aromatase inhibitor associated bone loss in postmenopausal women with early breast cancer receiving adjuvant letrozole: 36 months follow-up of ZO-FAST. General session #44, 2008, SABCS

27) Wnter MC, Holen I, Coleman RE : Exploring the anti-tumor activity of bisphosphonates in early breast cancer. *Cancer Treat Rev.* 34, 453-75, 2008

28) Sato K, Kimura S, Segawa H, *et al:* Cytotoxic effect of γδT cells expanded ex vivo by a third generation bisphosphonate for cancer immunotherapy. *Int J Cancer.* 116, 94-99, 2005
